# Advanced Strategies in Enhancing the Hepatoprotective Efficacy of Natural Products: Integrating Nanotechnology, Genomics, and Mechanistic Insights

**DOI:** 10.1021/acsbiomaterials.5c00004

**Published:** 2025-04-11

**Authors:** Jitendra Patel, Harekrishna Roy, Pavan Kuma Chintamaneni, Rukmani Patel, Raghvendra Bohara

**Affiliations:** † Datta Meghe College of Pharmacy, Datta Meghe Institute of Higher Education (Deemed to be University), Sawangi (Meghe), Wardha 442001, Maharashtra, India; ‡ Department of Pharmaceutics, Nirmala College of Pharmacy, Mangalagiri 522503, Andhra Pradesh, India; § Department of Pharmaceutics, GITAM School of Pharmacy, GITAM Deemed to be University, Hyerabad 502329, Telangana, India; ∥ Department of Chemistry, Bharati University Durg, Durg 491001, Chhattisgarh, India; ⊥ University of Galway, Galway H91 TK33, Ireland

**Keywords:** Hepatoprotective Agents, Natural Products, Liver Disorder, Nanotechnology, Genomics, Synergistic Strategies

## Abstract

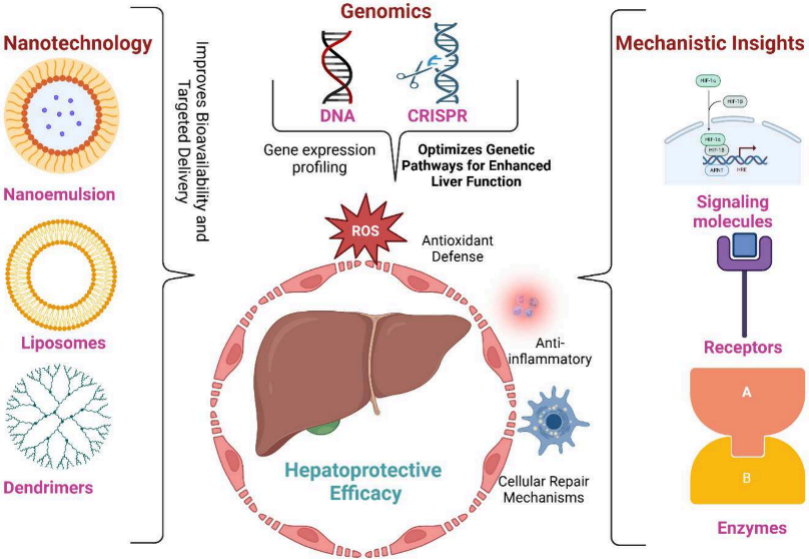

Liver disorders like hepatitis, cirrhosis, and hepatocellular carcinoma present a significant global health challenge, with high morbidity and mortality rates. Key factors contributing to liver disorders include inflammation, oxidative stress, and apoptosis. Due to their multifaceted action, natural compounds are promising candidates for mitigating liver-related disorders. Research studies revealed the antioxidant, anti-inflammatory, and detoxifying properties of natural compounds like curcumin, glycyrrhizin, and silymarin and their potential for liver detoxification and protection. With advancements in nanotechnology in drug delivery, natural compounds have improved stability and targetability, thereby enhancing their bioavailability and therapeutic efficiency. Further, recent advancements in genomics and an increased understanding of genetic factors influencing liver disorders and the hepatoprotective effects of natural agents made way for personalized medicine. Moreover, combinatorial therapy with natural products, synthetic drugs, or other natural agents has improved therapeutic outcomes. Even though clinical trials have confirmed the efficiency of natural compounds as hepatoprotective agents, several challenges remain unanswered in their translation to clinical practice. Therefore, it is logical to integrate natural compounds with nanotechnology and genomics to further advance hepatoprotection. This review gives an overview of the substantial progress made in the field of hepatoprotection, with specific emphasis on natural compounds and their integration with nanotechnology and genomics. This provides valuable insights for future research and innovations in developing therapeutic strategies for liver disorders.

## 1 Introduction

The liver plays a crucial role in our general well-being as it is a multipurpose organ that aids in detoxification and nutrient processing. When illnesses hinder their abilities, the repercussions are significant. Think about the experience of someone dealing with liver disease. Symptoms may be mild or nonexistent early, enabling the disease to advance undetected.^1^ At present, liver diseases result in the deaths of approximately 300,000–400,000 patients annually in China. The remaining challenges are low alertness to the severity of liver diseases and a low regimen rate for patients, particularly in the broad pastoral areas of China.^2^ Among various liver disorders, one of the most concerning is steatosis, which is characterized by an overabundance of hepatic fat linked to metabolic syndrome and obesity. Nonalcoholic fatty liver disease has emerged as the most prevailing liver disease, with a global impact on healthcare.^3^ Apart from metabolic causes, liver injury can also result from drug toxicity, which is a major concern in the United States and numerous European countries. Two studies have reported a rate of 2.7/100,000 individuals each year in prospective and retrospective drug-induced liver injury (DILI).^4^ Hepatic fibrosis is a critical component of the advancement of chronic liver disease, which conclusively results in cirrhosis and hepatocytic carcinoma. Cirrhosis has been primarily caused by the consumption of alcohol, hepatitis C, and hepatitis B on a global scale.^5^

In light of the escalating prevalence of liver illnesses, researchers are investigating natural substances such as flavonoids for their possible hepatoprotective properties. In light of the escalating prevalence of liver illnesses, researchers are investigating natural substances such as flavonoids for their possible hepatoprotective properties. Dietary flavonoids are crucial in expanding and extenuating pathological circumstances due to their antioxidant, hepatoprotective, and anti-inflammatory activities. Recent clinical outcomes have confirmed the valuable effects of main hepatoprotective flavonoids on liver diseases by inhibiting inflammation.^6^ Hepatoprotective properties were most frequently demonstrated in the plant classes Fabaceae and Asteraceae. The phytoconstituents demonstrated the maximum frequency of hepatoprotective action, including phenolic compounds, flavonoids, and alkaloids.^7^ This is a severe health concern, as liver damage can lead to obese liver, hepatitis, fibrosis, cancer, and cirrhosis. Numerous medicinal properties are associated with herbal compounds. Potent therapeutic activity has been demonstrated in liver injuries induced by a variety of toxicants and drugs by natural products and nutraceuticals.^8^ Numerous medicinal plants have historically been utilized for hepatoprotection, with 15 species from 9 families acknowledged for their therapeutic properties. Recently, the utilization of plants with medicinal values has been embraced as a result of the discovery of antioxidant constituents in plants. Medicinal plants are employed to alleviate various ailments; the list has been given in Table 1.^9^ The viability of cells is influenced by the concentration and time of the alcoholic extracts of *M. oleifera*.^10^

**Table 1:** Summary of Liver Diseases and Their Global Impact Statistics

S. N.	Liver Disease	Description	Global Impact	References
1	Hepatitis B	Viral infection causing liver inflammation	In 2019, an estimated 296 million individuals were afflicted with chronic hepatitis B, resulting in 820,000 fatalities each year.	Ghulam, F., Zakaria, N. *et al.*(11)
2	Hepatitis C	Viral infection leading to liver inflammation	In 2019, approximately 58 million individuals were diagnosed with chronic hepatitis C, resulting in 290,000 fatalities each year.	Khan, S., Nosheen, M. *et al.*(12)
3	Alcoholic Liver Disease	Liver damage due to excessive alcohol consumption	3 million deaths worldwide each year	Provan, L., Forrest, E. *et al.*(13)
4	Fatty Liver Disease (Nonalcoholic)	Accumulation of fat in the liver without alcohol.	Leading cause of chronic liver disease; 25% of the global population	Boccatonda, A., Andreetto, L.^14^
5	Liver Cirrhosis	Late stage of scarring (fibrosis) of the liver	Causes 1.32 million deaths annually worldwide	Badvath, D., Miriyala, A.^15^
6	Liver Cancer	Primary liver cancer	The fourth most common cause of cancer-related fatalities worldwide, resulting in approximately 830,000 deaths annually.	Hao, X., Fan, R.^16^
7	Autoimmune Hepatitis	Chronic inflammation of the liver due to the immune system	Rare, with an annual incidence rate of 1–2 per 100,000 individuals.	Puustinen, L., Barner-Rasmussen, N.^17^
8	Hemochromatosis	Iron overload disorder leading to liver damage	Affects 1 in 200–300 people of Northern European descent; can lead to cirrhosis and liver cancer	Kane, S., Roberts, C.^18^
9	Wilson’s Disease	Copper accumulation in the liver is a result of a genetic disorder	Rare, with an incidence rate of 1 in 30,000; can lead to severe liver damage if untreated	Jopowicz, A., Tarnacka, B.^19^

Curcumin is a polyphenol belonging to the diarylheptanoid group. The compound comprises a diferuloylmethane group connected to an α,β-unsaturated β-diketone (heptadiene-dione) group and contains two *o*-methoxy phenolic groups. Curcumin demonstrates a range of pharmacological characteristics, including anti-inflammatory, antioxidant, anticarcinogenic, antibacterial, antiviral, antimalarial, and hepatoprotective activities.^20^ In addition to curcumin, several phytochemical substances, including *M. koenigii* leaf extract, have demonstrated neuroprotective and hepatoprotective properties via diverse pathways. The neuroprotective effects of *M. koenigii* leaf extract and its active components are demonstrated through various mechanisms in the awareness and treatment of the liver and other organs.^21^ Triterpenoids, alongside polyphenols, have garnered interest for their significant hepatoprotective effects. Triterpenoids have emerged as the most significant categories of phytochemicals for hepatoprotective agents, as numerous naturally occurring triterpenoids have been documented to exhibit significant hepatoprotective effects.^22^ Among triterpenoids, bioactive isolates such as echinocystic acid, eclalbasaponin II, and schaftoside have exhibited hepatoprotective properties.^23^ Ayurvedic herbs, including andrographis, have a lengthy historical record of customary usage in treating liver dysfunction and disease and revitalizing the liver.^24^

The present review explores the assessment of advanced technology increasing the liver protection of herbal products, discussing genomic, mechanistic, and nanotechnology. Huge published articles focused on natural product’s hepatoprotective potentials, primarily phytochemical compositions, pharmacological investigation, and traditional uses. This study uniquely bridges the gap into advancements that enhance target delivery, bioavailability, and genomic insights. It highlights genomic insights applicable to personalized medicines, and novel molecular pathways by mechanistic evaluation. Furthermore, old articles often focus on isolation, but this study proposes a holistic approach by assessing the synergistic effects.

## 2 Mechanisms of Hepatotoxicity

Metabolomic analysis is an innovative and effective method for evaluating and forecasting drug-induced liver damage. A recent study examined HepG2 cells subjected to several chemicals to assess their hepatotoxic effects.^25^ Hepatocellular apoptosis occurs in cholestatic illness due to the body’s production of bile acids. Acetaminophen toxicity leads to the scavenging of superoxide by nitric oxide (NO), leading to the synthesis of peroxynitrite. This peroxynitrite leads to protein nitration and subsequent tissue damage—mitochondrial dysfunction, resulting in cell death, known as necrosis. Hepatotoxicity, resulting from various causes, remains a key factor for removing drugs from pharmaceutical research and clinical usage^25^

### 2.1 Common Causes and Types of Liver Damage

Non-Alcoholic Fatty Liver Disease (NAFLD) is the leading cause of chronic liver disease worldwide. The worldwide incidence of NAFLD has escalated significantly in conjunction with rising rates of obesity, type 2 diabetes, hypertension, and hypercholesterolemia. Individuals diagnosed with a fatty liver illness that is not caused by alcohol experience a higher risk of developing the condition of cardiovascular disease and cancer.^26^ The evolution of NAFLD and its related consequences can be alleviated with weight reduction, cessation of smoking, and dietary adjustments.^27^ Alcoholic steatohepatitis (ASH) and nonalcoholic steatohepatitis (NASH) are significant types of chronic liver disease, both defined by hepatic steatosis and inflammation. Mitochondrial dysfunction and inflammasome activation are pivotal in the advancement of ASH and NASH, leading to hepatic damage.^28^

Infectious liver damage is predominantly attributed to viral infections, such as Epstein–Barr virus, cytomegalovirus, and enterovirus, with *Mycoplasma pneumoniae* and bacterial infections also playing a substantial role. The most common causes of noninfectious liver damage were drug-induced hepatotoxicity, Kawasaki illness, and hereditary metabolic abnormalities. There were 31 instances of severe liver injury.^29^ Alcohol use disorder is the primary cause of liver disease in the Western world, leading to illnesses such as steatosis, steatohepatitis, fibrosis, cirrhosis, and hepatocellular carcinoma.^30^

Drug-induced liver injury is the most prevalent cause of liver damage and the most common reason for substance withdrawal. The manifestations of drug-induced liver injury are exceedingly varied, with certain patients remaining asymptomatic. Compared to the other antidepressants examined in this investigation, selective serotonin reuptake inhibitors are less likely to induce liver injury induced by drugs, particularly in patients with preexisting liver dysfunction.^31^

### 2.2 Cellular and Molecular Mechanisms of Hepatotoxicity

A recent study elucidated the hepatotoxicity induced by Aristolochic acids-I through single-cell RNA sequencing. The results indicate that the NFκB and STAT3 pathways mediate inflammatory responses in hepatocytes, while Apoptosis in liver sinusoidal endothelial cells is mediated by the STAT3/HMGB2. Furthermore, it demonstrates the infiltration of cytotoxic T- T-lymphocytes and the activation of macrophages and neutrophils by AAI, which suggests that AAI induces immune-inflammatory responses that result in liver injury.^32^ The initial comprehensive single-cell RNA sequencing analysis of Triclosan-induced hepatotoxicity demonstrates molecular changes in six liver cell types, constructs an interaction network, and emphasizes how Triclosan modulates various cell types to activate hepatic stellate cells, thereby promoting hepatotoxicity and fibrogenesis.^33^ The different way of molecular and cellular mechanisms has been briefed in Table 2. Key cellular events in acetaminophen-induced liver injury include malfunction and increased oxidative stress in the mitochondria. The potential therapeutic targets for autoimmune hepatitis are numerous biomolecules implicated in these processes.^34^

**Table 2:** Summary of Cellular and Molecular Mechanisms Involved in Hepatotoxicity

S.N.	Mechanism	Description	Key Features	References
1	Oxidative Stress	Imbalance between reactive oxygen species (ROS) production and antioxidant defenses	An overabundance of reactive oxygen species (ROS) results in the oxidation of lipids, damage to DNA, and oxidation of proteins, which in turn leads to harm to cells.	Pizzino, G., Irrera, N.^35^
2	Mitochondrial Dysfunction	Impairment of mitochondrial function and energy production	Decreased ATP production, increased ROS generation, and initiation of apoptosis or necrosis	Chen, X., Ji, Y.^36^
3	Inflammation	Activation of inflammatory pathways and immune responses	The occurrence of pro-inflammatory cytokines being released, Kupffer cells being activated, and the influx of leukocytes.	Cekici, A., Kantarci, A.^37^
4	Endoplasmic Reticulum Stress	Endoplasmic reticulum-mediated disruption of protein folding	Unfolded protein response (UPR) activation, leading to apoptosis if unresolved	Iurlaro, R., Muñoz-Pinedo, C.^38^
5	Cell Death Pathways	Activation of apoptotic or necrotic pathways	Involvement of caspases in apoptosis; loss of membrane integrity and release of intracellular contents in necrosis	Guha, L., Singh, N., Kumar, H.^39^
6	Lipid Accumulation (Steatosis)	Excessive accumulation of lipids in hepatocytes	Disruption of lipid metabolism, leading to fatty liver and progression to steatohepatitis	Zhang, X., Qin, J.^40^
7	Genotoxicity	Damage to genetic material (DNA) in liver cells	Mutations, chromosomal aberrations, and activation of DNA repair mechanisms	More, S., Bampidis, V.^41^
8	Disruption of Calcium	Imbalance in intracellular calcium levels	Activation of calcium-dependent enzymes, mitochondrial permeability transition, and cell death	Chebib, F., Sussman, C.^42^
9	Impaired Autophagy	Dysfunction in the degradation and recycling of cellular components	Accumulation of damaged organelles and proteins, contributing to cell injury	Hailfinger, S., Schulze-Osthoff, K.^43^
10	Fibrogenesis	The excessive accumulation of extracellular matrix proteins causes fibrosis.	Hepatic stellate cells are activated, leading to an increase in collagen production and the creation of scar tissue.	Lee, U., Friedman, S.^44^

**Figure 1:**
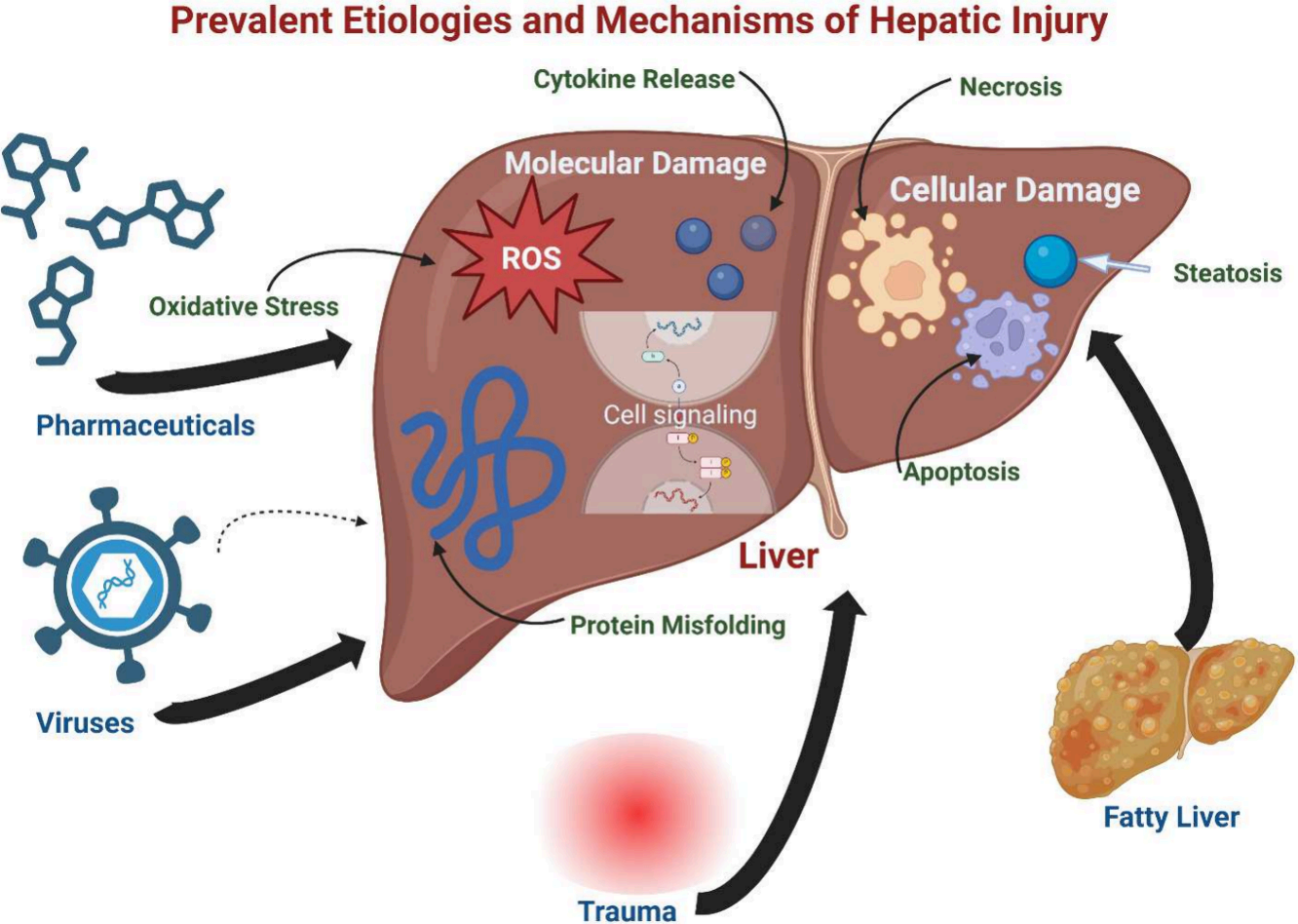
Prevalent etiologies and mechanisms of hepatic injury. This image depicts the diverse chemical, biological, physical, and metabolic etiologies of hepatic injury, encompassing pharmaceuticals, poisons, viruses, trauma, and fatty liver disease without alcohol. Hepatotoxicity processes are categorized into cellular damage (apoptosis, necrosis, steatosis) and molecular, resulting in liver injury via reactive oxygen species increase, protein misfolding, and cytokine release.

Phytochemicals restore the cellular antioxidant defense system, protecting against Acetaminophen-induced liver toxicity and mitigating necrotic cell death. This is accomplished by restricting oxidative stress, thus safeguarding against mitochondrial malfunction and inflammation.^45^ Similarly, hepatotoxicity induced by other drugs, such as Olanzapine, has also been explored. Olanzapine has been associated with severe hepatotoxicity and elevated liver enzymes in patients. The study assessed the cytotoxic impact of Olanzapine on newly isolated rat hepatocytes. It was discovered that the toxicity of Olanzapine is caused by excessive production of reactive oxygen species, leading to a collapse in mitochondrial potential, leakage in lysosomal membranes, depletion of GSH (glutathione), and lipid peroxidation. These effects occur before the disintegration of the cells.^46^ These findings highlight the diverse mechanisms of drug-induced liver damage, as summarized in Figure 1. The probable methods by which various intrinsic and idiosyncratic drugs cause liver damage encompass a range of molecular and cellular components in the processes of hepatotoxicity and cell death^47^

## 3 Natural Products with Hepatoprotective Properties

Naringenin is distinguished among hepatoprotective flavonoids for its antioxidant, anti-inflammatory, antifibrogenic, and anticancer characteristics. The preliminary protective activity of naringenin in liver illness inhibits the transdifferentiation of liver stellate cells, which results in reduced collagen synthesis and the reduction of oxidative stress and the transforming growth factor (TGF-β) pathway.^48^*Berberis aristata* comprises many hepatoprotective alkaloids, including as berberine, oxyberberine, karachine, and protopine, which enhance liver protection by regulating oxidative stress and inflammatory pathways. Resveratrol, which is also present in *Vaccinium myrtillus*, serves to mitigate hepatotoxicity.^49^ Thirteen polyphenolic and flavonoid molecules, such as epicatechin, catechin, kaempferol, quercitrin, quercetin-7-O-rhamnoside, isoquercitrin, anthocyanins, procyanidin A2, quercetin-3-O-rutinoside-7-O-a-L-rhamnoside, pelargonidin-3-O-glucoside, gallic acid, and hyperin, have been identified as important phytoconstituents in a variety of plant extracts, which have been shown to exhibit significant hepatoprotective properties. Sulfated polysaccharides that are highly active and isolated from seaweed possess various significant properties, including immunomodulatory, antitumor, neuroprotective, and hepatoprotective effects^50^

The hepatoprotective effects of fucoxanthin extract obtained from a commercially cultivated microalga called *Phaeodactylum tricornutum*. The HepG2 cell line was subjected to a 24-h investigation in culture, and its cytotoxicity was compared to that of methotrexate. The active compound demonstrated hepatoprotective effects against methotrexate in the HEPG-2 human hepatocyte cell line at a dosage of 0.25 mg/mL.^51^ Carrots are taproot vegetables rich in carotenoids, flavonoids, polyacetylenes, vitamins, and minerals, all exhibiting various nutritional and hepatoprotective properties.^52^ Figure fruits’ functional properties and technological capabilities as a dietary supplement are attributed to their broad range of bioactive compounds, including phenols, carotenoids, flavonoids, and vitamin C, which are responsible for their liver protection impacts.^53^ One of the most significant medicinal plants is *Cassia fistula* L, which reveals a variety of pharmacological properties, such as antioxidant, antimicrobial, anti-inflammatory, antidiabetic, antitumor, and hepatoprotective properties, among others.^54^

### 3.1 Overview of Various Natural Products and Their Sources

This study systematically analyzes 15 key phytoconstituents, detailing their plant sources, extraction methods, hepatoprotective assays, and proposed mechanisms of action, as summarized in Table 3. This text provides a succinct analysis of nine potential hepatoprotective substances derived from natural sources, detailing their chemical composition and the mechanisms by which they protect the liver.

**Table 3:** Hepatoprotective Agents from Natural Sources

S.N.	Natural Source	Major constituents	Mechanism of action	References
**1**	Silymarin (family: Asteraceae)	silybin, isosilybin, silydianin, and silychristin	Manipulation of hepatic biochemical indicators, both enzymatic and nonenzymatic.	Soleimani, V., Delghandi, P.^55^
**2**	Glycyrrhizin (family: Leguminacae)	glycyrrhetinic acid, beta-sitosterol, hydroxycoumarins, and flavonoids	increasing antioxidant defense in hepatic cell and as anti-inflammatory agent	Salminen, H., Kasapoğlu, K.^56^
**3**	Andrographolide and neoandrographolide (family: Acanthaceae)	The compounds mentioned include neoandrographolide, 14-deoxy-11-dehydroandrographolide, 14-deoxy-11-oxoandrographolide, deoxy-andrographolide, andrographolide, and andrographine.	inhibits inflammation, angiogenesis, and fibrosis	Raman, S., Murugaiyah, V.^57^
**4**	Kutkoside and Picroside	The compounds mentioned are kurkoside, apocynin, drosin, cucurbitacin glycoside, and the iridoid glycosides picroside 1, 2, and 3.	membrane stabilizing, hypolipidemic and antioxidant properties	Gaikwad, P., Bhope, S.^58^
**5**	Curcumin (family: Zingiberaceae)	Curcumin, demethoxycurcumin, and bisdemethoxycurcumin	The activity of antioxidants and the stimulation of phase 2 detoxifying/antioxidant enzymes, specifically HO-1.	Hewlings, S., Kalman, D.^59^
**6**	Phyllanthin and hypophyllanthin (family: Euphorbiaceae)	The compounds present in the substance are alkaloids, astragalin, brevifolin, ellagitannins, amariin, repandusinic acid, phyllanthusiin D gallocatechins, geraniin, hypophylanthis, lignans, nirutin, phyllanthin, and phyllanthenol.	liver-protective and detoxifying action	Nasrulloh, R., Rafi, M.^60^
**7**	Berberine (family: Berberidaceae)	The compounds included are berberine, oxyberberine, berbamine, aromoline, karachine, and oxycanthine.	suppress oxidative stress and attenuates apoptosis	Och, A., Podgórski, R., Nowak, R.^61^
**8**	Embelin (family: Myrsinaceae)	embelin, christembine, quercitol, and resin	Pathway involving the scavenging of free radicals and the peroxidation of lipids.	Ko, J., Lee, S.^62^
**9**	Resveratrol, *Vitis labrusca*, commonly known as “grapes”	trans-3,5,4′- trihydroxystilbene	Altering the activity of nuclear transcription factors Nrf2 and NF-κB and reducing the expression of HO-1 and iONS genes.	Lambert, C., Lemaire, J.^63^

Over 350 natural triterpenoids have been found to possess hepatoprotective effects, documented in over 50 plant species across 27 plant families. Many of these triterpenoids exhibited substantial hepatoprotective properties in response to different exogenous stimuli and were considered potential hepatoprotective agents for clinical application. Plants of the *Hypericum L*. genus are found throughout the globe. These plant species are currently employed in various traditional medical systems to treat liver disease^64^

### 3.2 Traditional Uses and Ethnopharmacology

Phytochemical analysis of *M. spicata’s* numerous parts identified 35 chemical constituents, including phenolic acids, flavonoids, and lignans.^65^ Diverse phytochemicals (volatile: terpenoids, fatty acids, phenols, etc.; nonvolatile: flavonoids, flavanones, chalcones) with antioxidant, antimicrobial, anti-inflammatory, hepatoprotective, and other biological activities, including potential against COVID-19, are present in *B. balsamifera*.^66^ 208 chemical constituents such as sesterterpenoids, terpenoids, diterpenoids, and flavonoids were identified from the Scutellaria genus. The biological activities of extracts and compounds are diverse, including antioxidant, anticancer, anti-inflammatory, antimicrobial, and effects on cardiovascular, cerebrovascular, and hepatoprotective conditions.^67^. The anthelmintic, antiarthritic, anticonvulsant, anti-inflammatory, antioxidant, antimicrobial, antidiabetic, and hepatoprotective properties of *Carissa spinarum* L. (Apocynaceae) were disclosed.^68^ A comprehensive meta-analysis summarizing major hepatoprotective active compounds, including those from *Carissa spinarum* and other medicinal plants, is presented in Table 4.

**Table 4:** Meta-analysis Data for Key Hepatoprotective Compounds

Study Reference	Compounds	Model Used	Key Findings
Harish, R., Shivanandappa, T. et al.^72^	*Phyllanthus niruri*	Carbon tetrachloride (CCl4) - induced	Antioxidant Activity, Hepatoprotective Activity.
Jain, K., Majee, C. et al.^144^	3, 19 (4-bromobenzylidene)	Paracetamol induced hepatotoxicity	Hepatoprotective Activity.
Patel, J. et al.^188^	*Terminalia coriacea*	Carbon tetrachloride (CCl_4_) - induced	Hepatoprotective Activity.
			Anti-inflammatory
Sun, J. et al.^73^	Purple sweet potato polysaccharides	Carbon tetrachloride (CCl_4_) - induced	Potential antioxidant activity and protective effect.
Cheng, M. et al.^133^	*Glycyrrhiza uralensis*	d-galactosamine-induced toxicity	Hepatoprotective Activity.
Chattopadhyay, R. et al.^90^	*Azadirachta indica*	Paracetamol induced hepatotoxicity	Hepatoprotective Activity.
Huang, Z. Q. et al.^34^	Quercetin 7-rhamnoside	H_2_O_2_-induced oxidative damage cellular model	Natural antioxidants
Feng, X. H. et al.^91^	*Terminalia chebula* fruit	*tert*-butyl hydrogen peroxide (t-BHP), acetaminophen (APAP), and CCl_4_,	Hepatoprotective effects
Cao, S.^141^	Chinese teas	Acute alcohol-induced liver injury.	antioxidant and hepatoprotective actions
Sharma, S. K.^99^	*Chlorophytum borivilianum* root	Arsenic-induced toxicity	

**Figure 2:**
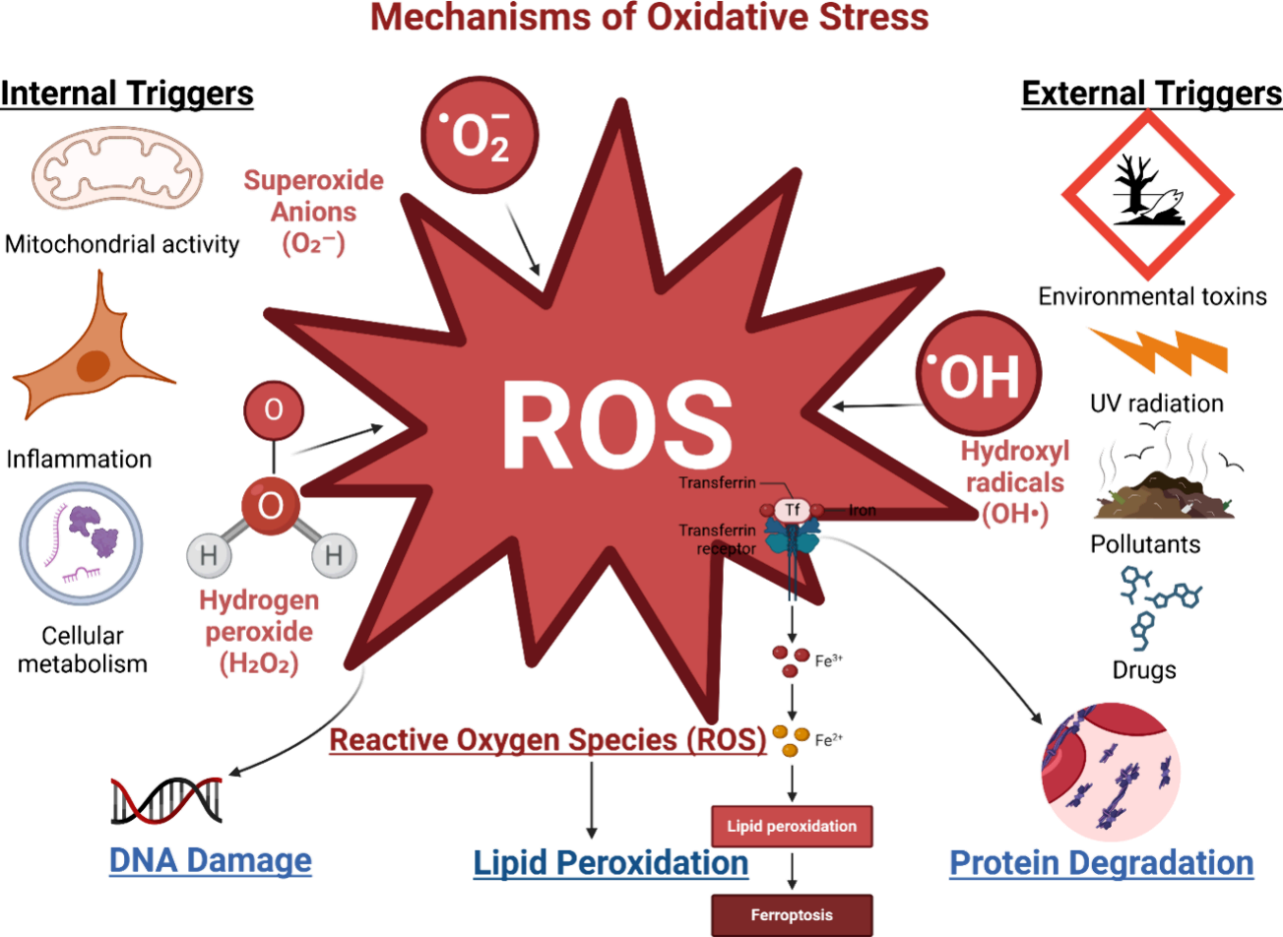
Mechanisms of oxidative stress. The graphic illustrates oxidative stress caused by external and internal variables, resulting in the creation of reactive oxygen species (superoxide anions, hydrogen peroxide, hydroxyl radicals). Cellular defenses, comprising enzymatic (SOD, CAT, GPx) and nonenzymatic systems, mitigate reactive oxygen species (ROS). DNA repair enzymes, protein degradation, and lipid peroxidation restoration mitigate oxidative damage, safeguard against chronic illnesses, and preserve cellular homeostasis.

Iridoid and phenylpropanoid glycosides are identified in phytochemical analyses of *S. grosvenorii*. Triterpenoids, flavonoids, and amino acids are among the isolated compounds. Hypoglycemic, Antioxidant, immunologic, sputum-reducing, antitussive, and hepatoprotective properties are exhibited by *S. grosvenorii* and its constituents.^69^ The collection of 233 compounds of various categories, including flavonoids, alkaloids, essential oils, fatty acids, terpenoids and phenols, has been facilitated by *A. vasica*. It is an excellent source of potential phytopharmaceutical compounds that reveal a variety of pharmacological activities, such as antifungal, antibacterial, hepatoprotective, abortifacient, antiulcer, antiviral, thrombolytic, anti-inflammatory, hypoglycemic, antioxidant, antitubercular and antitussive properties.^70^ Homoisoflavonoids, flavonoids, alkaloids, esters, organic acids, lignans, catecholamines, terpenoids, sterols, and cerebrosides are abundant in *Portulaca oleracea* L. It demonstrates several pharmacological activities, including hepatoprotective actions, due to its abundant presence of homoisoflavonoids, flavonoids, and alkaloids.^71^ The mechanism of oxidative stress is revealed in Figure 2.

## 4 Mechanisms of Action of Natural Hepatoprotective Agents

The antioxidant properties of phytoconstituents are the primary mechanism by which they alleviate several disease pathways. This is achieved by enhancing the antioxidant protection system of cells, scavenging free radicals, reducing peroxidation of lipids, improving anti-inflammatory activity, and further saving hepatic cell injury.

### 4.1 Antioxidant Activity

Research revealed that the aqueous and methanolic extracts of *P. niruri* exhibited strong inhibitory effects on the in vitro production of microsomal lipid peroxidation by Fe2+ and ascorbate. Research findings indicate that fruit and leaf extracts exhibit antioxidant properties. The fruit and leaf extracts in water were more effective in suppressing superoxide (ROS) *in vitro* than methanolic extracts. Additionally, these extracts demonstrated significant DPPH scavenging activity.^72^*Hypericum japonicum* has been a part of Chinese traditional medicine for ages to address hepatitis and cholestasis. Studies revealed the presence of a flavonoid quercetin-7-rhamnoside (Q7R), a natural antioxidant reservoir that has the potential to treat liver injuries. Polysaccharides, namely PSWP, PSAP-1, and PSAP-2, were extracted sequentially using hot water from sweet potato tubers. The polysaccharides were evaluated for their antioxidant in vitro and hepatoprotective activities in vivo in CCl_4_-induced hepatotoxicity models of rats.^73^*Ampelopsis grossedentata* is widely known for its medicinal benefits and is part of Chinese and Indian traditional medicine. Extracts from this plant were shown to have antioxidant and hepatoprotective activities.^74^ Phenolic extracts of *Halimeda opuntia* (Linnaeus) Lamouroux, a waterless algae, have shown hepatoprotective and antioxidant activity. They were shown to regulate the liver’s oxidative state and antioxidant enzymes.^75^*Fimbristylis miliacea,* a grasslike herb, has high concentrations of many polyphenols, including caffeic acid and kaempferol. These compounds were reported to have antioxidant and hepatoprotective properties^76^

### 4.2 Anti-inflammatory Effects

Ethanolic extracts of *Mahonia oiwakensis* Hayata were reported to have hepatoprotective activity by showing anti-inflammatory activity. This activity is related to a reduction in the concentration of nitric oxide and malondialdehyde (MDA) in the liver, along with antioxidant activity regulation.^77^ Isorhamnetin, a rflavonoid derived from leave and fruits of *Hippophae rhamnoides* L. showed hepatoprotective activity. This can be attributed to its lipid lowering, antioxidant, anti-inflammatory properties.^78^ Coadministration of acetylsalicylic acid and curcumin showed potential interactions. It was discovered that curcumin exhibits an enhanced antioxidant capacity. Utilizing curcumin as a supplementary treatment alongside acetylsalicylic acid for extended liver care.^79^ One study explores the hepatoprotective effects of exopolysaccharides (EPS) derived from the mushroom *Pleurotus geesteranus* in relation to alcohol-induced liver damage in rodents. The compound was determined to be a heteropolysaccharide with an α-glycosidic bond. This product shows promise as a functional food supplement or natural medication for preventing liver injury.^80^ In a rat model, the effects of quercetin (QT) on inflammation and systemic toxicity were examined. It demonstrated hepatoprotective and anti-inflammatory properties in this model.^81^

### 4.3 Modulation of Detoxifying Enzymes

There may be a lack of scientific evidence supporting the use of *Persea americana* in treating certain ailments, such as hypertension and diabetes. The extracts’ concentration positively correlates with the inhibitory action of *P. americana* on alpha-glycosidase and alpha-amylase enzymes. Consuming *P. americana* results in the accumulation of traits that inhibit enzymes, safeguard the liver, and function as antioxidants.^82^ One study explores the hepatoprotective effects of alkali- and enzyme-extractable polysaccharides from *Dictyophora indusiata* (Al-DPS and En-DPS) in mice with hyperlipidemia. The Al-DPS and En-DPS are innovative compounds with promising applications in treating hyperlipidemia and as hepatoprotective medicines.^83^

The impact of dietary Aloe vera on the activities of hepatoprotective enzymes, antioxidants, and plasma lipid profile. It is appropriate for enhancing the activities of hepatoprotective enzymes, antioxidants, and plasma lipid profile.^84^ Women experience more liver damage than males as a result of chronic alcohol consumption. The liver impairment in female rats that were administered ethanol in a diet that contained fish oil was more severe than that in male rats. Significant amounts of liver enzymes were secreted into the bloodstream.^85^ The chip was equipped with three hepatoprotectants, along with the introduction of acetaminophen as a toxic substance. The mechanisms of action of these hepatoprotectants were discovered through the observation that bifendatatum mainly reduced the secretion of alanine transaminase, tiopronin primarily reduced the secretion of lactate dehydrogenase, and glycyrrhizinate primarily reduced the secretion of aspartate transaminase^86^

### 4.4 Regulation of Apoptotic Pathways

Acute liver injury (ALI) is a serious and potentially fatal condition characterized by the death of liver cells (hepatocytes) caused by an overwhelming presence of oxidative stress and inflammation. Myricetin is a bioflavonoid found in certain berries, such as blueberries and strawberries. It demonstrates beneficial properties for reducing inflammation, combating oxidative stress, and preventing cell death.^87^ Various cellular processes, such as apoptosis, are regulated by protein kinase C. The primary function of novel PKCs is to participate in the apoptotic process. The antioxidant ellagic acid demonstrates hepatoprotective effects. the regulation of PKC-mediated apoptosis in the liver of lymphoma-bearing mice by the effect of ellagic acid on novel and atypical isozymes of PKC.^88^ The progression of nonalcoholic steatohepatitis has been linked to the role of hepatocyte apoptosis and inflammation. The activation of Fas receptor signaling appears to accelerate these pathways due to the overproduction of reactive oxygen species (ROS). Consequently, Investigated the hepatoprotective properties of crocin as a potent free radical scavenger in the context of oxidative injury that results in the development of nonalcoholic steatohepatitis.^89^

### 4.5 Other Relevant Mechanisms

**Figure 3:**
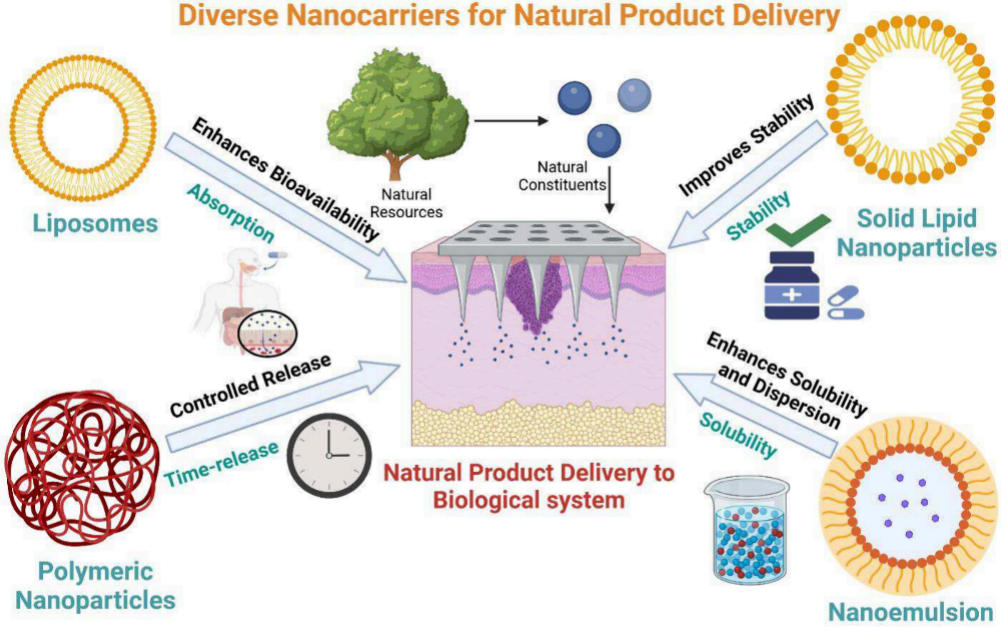
Diverse nanocarriers for natural product delivery. The figure illustrates nanocarriers such as polymeric nanoparticles, liposomes, solid lipid nanoparticles, and nanoemulsion. Liposomes enhance bioavailability. Polymeric nanoparticles offer controlled release, while solid lipid nanoparticles improve stability.

The hepatoprotective effects of *Azadirachta indica* leaf extract in paracetamol-induced hepatotoxicity result from a combination of enzyme-modulating, anti-inflammatory, and antioxidant activities. These mechanisms collaborate to mitigate oxidative stress, improve detoxification, prevent lipid peroxidation, reduce inflammation, and stimulate liver cell regeneration, thereby reversing the detrimental effects of paracetamol on the liver.^90^*Terminalia chebula* is a plant that has been extensively studied in scientific research. Retz. fruit is widely recognized in traditional medicine for its potential benefits in treating liver ailments. Scientific research has shown that Chemilinic acid (CA), which is a prominent chemical found in *T. chebula* fruit, has significant hepatoprotective properties. The study demonstrated that natural substances can activate Nrf2 and significantly protect the liver.^91^*Phytochemicals elicit* a multifaceted approach to show hepatoprotective activity. These include managing oxidative stress, reducing live inflammation, upregulating detoxification pathways, inhibiting lipid peroxidation, and improving cell repair and regeneration. These multifaceted mechanisms make them valuable in treating and preventing liver-related disorders.^92^ Given the multifaceted mechanisms of hepatoprotective phytochemicals, novel drug delivery approaches such as nanocarriers are being explored to enhance their efficacy. Different types of nanocarriers are displayed in Figure 3.

## 5 Nanotechnology in Enhancing Hepatoprotective Efficacy

Recent advancements in nanotechnology have transformed the field of drug delivery by enhancing the bioavailability of hepatoprotective phytochemicals. Further, nanotechnology enhances these phytochemicals’ targetability, therapeutic potential, and controlled delivery. This precise approach maximizes the therapeutic potential and reduces toxicity, thereby protecting liver cells from damage. As research in nanotechnology advances, it paves the way for treating advanced liver disorders.

### 5.1 Overview of Nanotechnology Applications in Medicine

Nanotechnology has considerably progressed medicine by improving drug delivery, especially in hepatoprotection. Nanomedicine approaches enhance the solubility, stability, and bioavailability of hepatoprotective phytochemicals, facilitating targeted liver delivery and reducing systemic toxicity. Pharmaceutical companies worldwide are leveraging nanotechnology and phytomedicine to enhance productivity, design, and structure efficiency. It has also brought about advancements in larger-scale production units in sectors like automobile manufacturing, civil engineering, and environmental management^93^ Nanomedicine is the term used to describe the application of nanotechnology in the fields of medicine and healthcare. It has been employed to combat some of the most prevalent diseases, such as cancer and cardiovascular diseases.^94^ In the United States, there has been a significant increase in the number of medicinal products containing nanomaterials submitted over the past two decades. The primary product category includes liposomal formulations primarily developed for cancer treatments. Out of the total, 65% fall under the category of experimental new medications, while 17% are new drug applications and 18% are abbreviated new drug applications. Around 80% of the products possess an average particle size of 300 nm or lower.^95^ Nano radiopharmaceuticals, also termed radiolabeled nanomaterials, leverage nanoparticles’ distinct physical and functional properties and show the potential to improve the imaging of human diseases.^96^ The potential to revolutionize the field of medicine and healthcare services lies in the emerging field of nanotechnology, which encompasses advancements in nanomedicine, nano implants, nano biosensors, and the Internet of nano things. In recent years, there has been a notable shift in organ transplantation, making it a more dependable option for individuals dealing with end-stage organ failure. The fusion of nanotechnology and transplantation introduces a fresh and groundbreaking perspective to transplantation medicine.^97^ The application and advancement of nanotechnology in kidney and islet transplantation involve a wide range of areas, including the preservation of renal tissue before transplantation, the creation of synthetic biological islets, organ imaging, and targeted drug delivery.^98^

### 5.2 Types of Nanocarriers Used for Natural Products

Nanocarriers, including liposomes, solid-lipid nanoparticles, nanoemulsions, and nanostructured lipid carriers, have been extensively utilized to augment phytochemicals’ bioavailability and hepatoprotective effectiveness, facilitating prolonged release and enhanced liver targeting. Different types of nanocarriers are displayed in Figure 3. Essential oils become unstable when directly added to a food product.^99^ The potential of plant-derived exosome-like nanoparticles to modulate immune responses is underscored by their emerging function in immune regulation and periodontitis treatment. By effectively targeting inflammatory pathways and enhancing tissue regeneration, these nanoparticles provide innovative therapeutic strategies for managing periodontal diseases.^100^ Bioactive compounds are enriched in products that have the potential to produce a diverse array of health benefits. Most BCs are hydrophobic and susceptible to environmental factors; consequently, encapsulation is implemented to solve these challenges. bovine serum albumin is the most frequently employed for producing BC-loaded nanocarriers.^101^ Nanomaterials with various properties have been implemented to enhance the therapeutic efficacy of these products. Special attention has been given to the ecological synthesis of nanocarriers loaded with these products or their extracts.^102^ An overview of nanocarriers emphasizes their importance in drug delivery, providing enhanced bioavailability, targeted delivery, and controlled release. These sophisticated systems are a promising instrument in modern medicine for various treatments, as they reduce side effects and improve therapeutic efficacy^103^

### 5.3 Benefits of Nanotechnology in Drug Delivery and Bioavailability

Significant research is being undertaken on drug delivery strategies that can improve the bioavailability of drugs, reduce side effects, and improve diagnosis. Nanoformulation systems like lipid and polymeric nanoparticles, ethosomes, and cyclodextrins have improved the bioavailability of Phytocannabinoids, which in their native form have limited delivery.^104^ Due to their unique properties, gold nanoparticles are used in medical research to diagnose several malignancies, drug delivery, and disease treatment. Vitamins such as vitamin D and K are beneficial in treating osteoarthritis, cardiovascular diseases, and cancer. However, their delivery in naïve form is very limited. Organic and inorganic carriers have been shown to improve their bioavailability and therapeutic activity by improving cellular transport.^105^ Nanotechnology has also emerged as a promising system for overcoming the challenges associated with the delivery of drugs by nasal route. The noninvasive intranasal route, ease of administration, presence of high vasculature, presence of the porous epithelial barrier, and ease of administration make it a highly suitable route for drug delivery. The difference between traditional and nanotechnology delivery of drugs is compared in Table 5. Furthermore, the nasal route also acts as a route of delivery of drugs to the brain via olfactory epithelium, thereby reducing peripheral side effects and improving brain bioavailability.^106^ In addition to enhancing medication delivery methods, including intranasal and oral administration, nanotechnology is vital in theranostics, which combines diagnosis and treatment. Advanced nanomaterials are being investigated to detect liver illness and deliver targeted hepatoprotective drugs, potentially transforming the treatment of liver cancer and fibrosis.^107^

**Table 5:** Comparison of Conventional vs Nanotechnology-Enhanced Delivery Systems

S.N.	Aspect	Conventional Delivery Systems	Nanotechnology-Enhanced Delivery Systems	References
1	Drug Solubility	Limited solubility for many drugs	Enhanced solubility and bioavailability of poorly soluble drugs	Khadka, P., Ro, J.^108^
2	Absorption	Variable and often inefficient absorption	Improved and more consistent absorption	Azman, M., Sabri, A.^109^
3	Targeted Delivery	Nonspecific, affecting both diseased and healthy tissues	Targeted delivery to specific cells or tissues, reducing off-target effects	Liang, Y., Duan, L.^110^
4	Bioavailability	Often low due to first-pass metabolism and degradation	Increased bioavailability due to protection from degradation and improved absorption	Manach, C., Scalbert, A.^111^
5	Side Effects	Higher incidence of side effects due to nonspecific distribution	Reduced side effects due to targeted delivery and controlled release	Due, A.^112^
6	Dose Frequency	Frequent dosing required	Potential for reduced dosing frequency due to sustained and controlled release	Jacob, S., Nair, A., Morsy, M.^113^
7	Drug Stability	Limited stability, susceptible to degradation	Enhanced stability and protection from environmental factors	Ashutosh, K. Y., Abhishek, Y.^114^
8	Therapeutic Efficacy	Lower efficacy due to poor targeting and bioavailability	Enhanced efficacy through improved targeting and bioavailability	Gubae, K., Mohammed, H.^115^
9	Cost	Generally lower initial cost but may require higher doses	Potentially higher initial cost but more cost-effective in the long run due to lower doses and reduced side effects	Mattingly, T., Weathers, S.^116^
10	Formulation Flexibility	Limited options for formulating poorly soluble or unstable drugs	High flexibility in designing formulations to enhance solubility, stability, and targeting	Pombo, D., Martinez-Rico, J.^117^
11	Particle Size,	Usually >1 μm (often 1–100 μm)	Typically, <200 nm (often 10–100 nm)	Khadka, P., Ro, J.^108^
12	Zeta Potential	Poorly defined, may aggregate	Generally, −30 mV to +30 mV (stable dispersion)	Malik, S., Muhammad, K., Waheed, Y.^93^

### 5.4 Case Studies and Examples of Nanotechnology-Enhanced Hepatoprotective Agents

**Figure 4:**
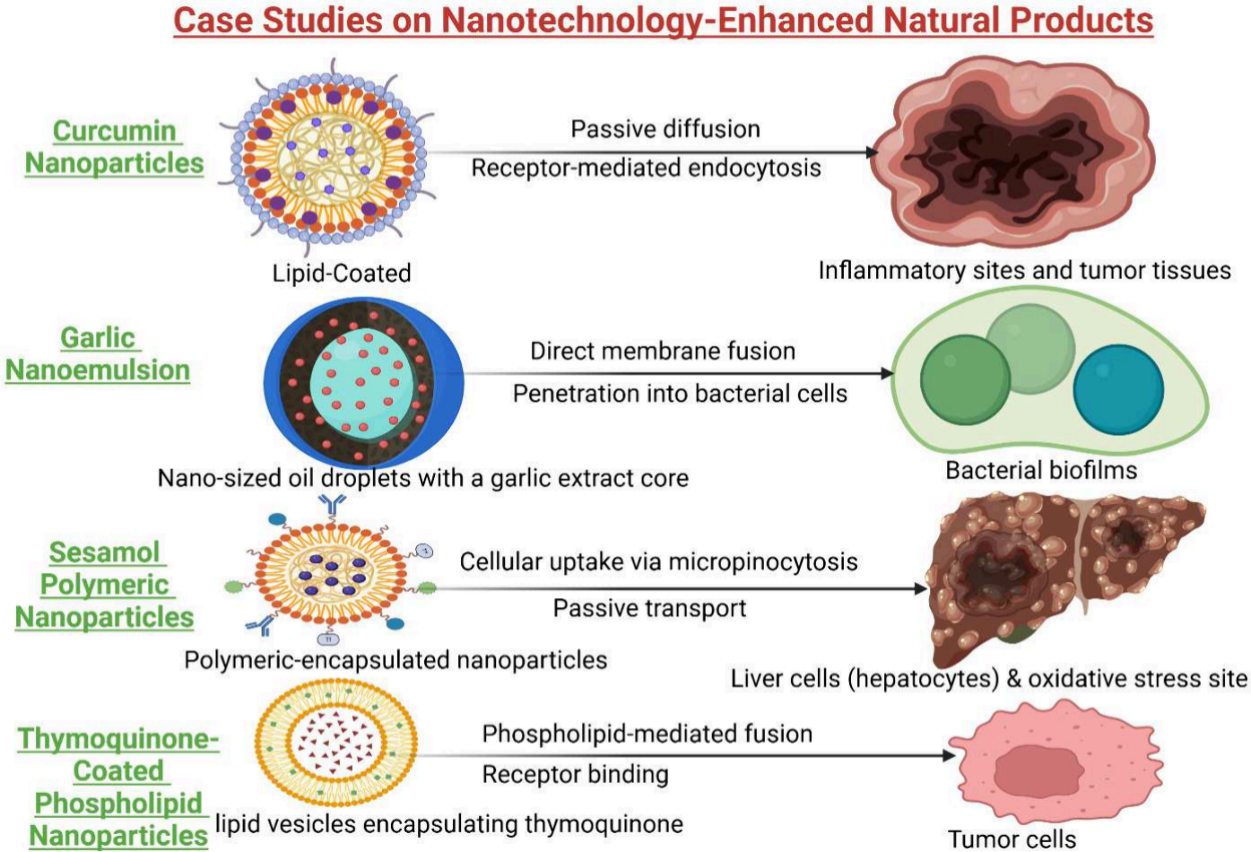
Case studies on nanotechnology-enhanced natural products. Curcumin nanoparticles enhanced anti-inflammatory and anticancer efficiency, garlic nanoemulsions augmented antibacterial properties, and sesamol polymeric nanoparticles improved antioxidant stability and bioavailability. Thymoquinone-coated phospholipid nanoparticles exhibited enhanced therapeutic efficacy in cancer treatment, underscoring the significance in improving the solubility, stability, and bioefficacy of natural chemicals for therapeutic progress.

Curcumin is a yellow phenolic component in several plants, with turmeric being one of the major sources. This natural compound has been extensively studied for its innumerable health benefits. It is found to possess strong antioxidant, anti-inflammatory, and antimicrobial characteristics. Further, it also has anticancer and neuroprotective effects. Nanoformulations of curcumin were studied for their efficiency in cancer cell lines, animal models, and clinical trials.^118^ Bioactive compounds present in garlic extract and garlic essential oils have been shown to have hepatoprotective effects. Several techniques like spray drying, complex coacervations, and nanotechnology were used to produce nanoemulsions, nanophytosomes, and nanoliposomes, which have been shown to significantly enhance the hepatoprotective activity of garlic bioactive compounds and improve stability.^119^ Sesamol, a phytoconstituent from sesame oil, was shown to have antioxidant potential and is efficient in neutralizing hydroxyl radicals. The polymeric nanoparticles of Sesamol were developed to address the drawbacks of traditional methods. The physicochemical properties, along with the biological efficiency to safeguard the liver in experimental animals, were carried out, showing an improved efficiency of the polymeric nanoparticles over traditional delivery methods.^120^ Similarly, lipid-based delivery systems of Thymoquinone were developed to improve the bioavailability and hepatoprotective activity. The developed lipid nanoparticles were observed to have a spherical shape with a particle size of less than 100 nm and PDI within an acceptable range. Further, the nanoparticles showed controlled drug release.^121^ The bioavailability, stability, and shelf life of these sensitive constituents were improved through encapsulation. The oral administration of nano beta-glucan capsules at concentrations of 100 and 200 mg for four consecutive days suggests that they act as a hepatoprotective agent.^122^ Case study on different phytoconstituents responsible for hepatoprotection listed in Figure 4.

## 6 Genomic Approaches in Hepatoprotection

In order to comprehend the mechanisms of liver disease and identify protective genes, genomic approaches in hepatoprotection employ sophisticated techniques such as gene editing, transcriptomics, and proteomics. These methods facilitate personalized medicine by customizing treatments according to genetic profiles, improving the efficacy of hepatoprotective therapies, and establishing the foundation for new therapeutic targets.

### 6.1 Role of Genomics in Understanding Liver Diseases

Genomic studies have been utilized to assess the hepatoprotective properties of natural compounds. One study evaluated the impact of Zamzam water (alkaline) on the integrity of genomic DNA in rodent liver tissues, indicating its potential protective significance for the liver. The normal structural and functional capacity of the hepatocytes is restored by Zamazam water. The experimental model of hepatic alterations in rodents exhibited a protective effect in both the molecular and histopathological results. This suggests that Zamazam water may be used as a hepatoprotective agent in the diet of patients with hepatopathies.^123^ The in vivo hepatoprotective and antioxidant properties of the seed extracts of *Cleome viscosa* Linn. (Capparaceae). The genomic DNA nicking assay extract was employed to evaluate the crude seed extract’s potential for DNA damage protection. The hepatoprotective activity of the plant extract was studied in CCl4-induced liver injury models of Wister albino rats.^124^ Root extracts of dandelion were used in clinical settings for liver cleansing and were evaluated in CCl4-induced hepatotoxicity models of Wister albino rats and measured for genomic DNA integrity.^125^ Hypomethylation of genomic DNA was found to be rectified by Betaine, a methyl donor. Supplementation with betaine to rodents in adolescence can safeguard them from nonalcoholic fatty liver induced by a high-fat diet.^126^ Phytoconstituents from *Cynara cardunculus* were found to have antioxidant, hepatoprotective, antihyperlipidemic, and antibacterial properties. Many works have been reported to elucidate the biosynthetic pathways of these chemicals via genomic and biochemical methods.^127^

### 6.2 Genetic Factors Influencing Hepatoprotection

Hepatoprotection is affected by genetic variables, including differences in antioxidant response genes, polymorphisms in detoxification enzymes, and differential expression of cytokines and growth factors. Recent studies have emphasized the significance of exosomes—membrane-derived nanovesicles—as crucial mediators in genetic transmission, affecting liver regeneration and tolerance to pollutants.

Exosomes are membrane-derived nanovesicles that are released by a variety of cells and have a dimension that ranges from 40 to 160 nm. They contain diverse payloads, such as coding RNAs, noncoding RNAs, proteins, and lipids. Exosomes have been identified as intercellular communication agents in recent studies. They have a crucial function in the physiological or biological processes of acute or chronic liver issues, as they horizontally transmit genetic bioinformation from donor cells to adjacent cells.^128^ Much research has been dedicated to utilizing zebrafish (*Danio rerio*) as a model organism to investigate the mechanisms governing hepatic growth during liver development and regeneration. Zebrafish possess distinct advantages when compared to other vertebrates. These advantages include the remarkable ability to conduct in vivo imaging at a cellular level and the opportunity to perform extensive chemical and genetic testing on a large scale.^129^ Steatosis exacerbates hepatic ischemia/reperfusion injury, which is a potential danger in fatty liver transplants. Adenosine receptors are highly promising therapeutic targets. The activation of A2AR through CGS21680 protects against injury by inhibiting ASK1/JNK through PI3K/Akt, whereas the activation of A1R through CCPA exacerbates injury. These results indicate that A2AR agonists could effectively prevent injury in obese liver surgeries.^130^

The translocation of cytochrome P450 2E1 in hepatocytes and an increase in GDF15 secretion result from chronic alcohol consumption, which elevates catecholamine levels. This increases the expression of ADRB2 in Kupffer cells, thereby reducing inflammation and promoting apoptosis. This highlights a novel gut-liver neuro-metabolic-immune mechanism for hepatoprotection, as the catecholamine/GDF15 axis protects against alcohol-associated liver disease.^131^ Genetic and environmental factors influence liver diseases. Interleukin-6 (IL-6) is frequently perceived as detrimental; however, it actually promotes liver regeneration and mitigates inflammation. The therapeutic effectiveness of IL-6 in treating liver diseases is emphasized by its ability to promote cell growth, stimulate blood vessel formation, and enhance metabolism, while also reducing cell death and oxidative stress^132^

### 6.3 Genomic Tools and Technologies Used in Research

#### 6.3.1 Next-Generation Sequencing (NGS)

Facilitates the rapid, high-throughput sequencing of entire genomes, offering comprehensive insights into genetic variations and mutations.

Next-generation sequencing (NGS) has transformed life science research and clinical diagnostics, especially detecting genetic variants linked to liver illnesses. NGS facilitates high-throughput sequencing of complete genomes, offering extensive insights into genetic alterations and polymorphisms associated with hepatoprotection. Nonetheless, despite its benefits, NGS is limited by elevated error rates, which might affect the precise identification of uncommon single nucleotide polymorphisms (SNPs) and mutations in genomic research.^133^ ABI SOLiD, Roche 454, and Illumina/Solexa are examples of next-generation sequencing platforms that have stimulated research in whole-genome shotgun assembly algorithms. Since 2005, the development of specialized assembly software has been driven by the fact that these platforms offer reduced read lengths, higher coverage, and varied error profiles in comparison to Sanger sequencing^134^

#### 6.3.2 CRISPR-Cas9

A potent gene-editing instrument that enables the implementation of precise modifications to DNA, thereby facilitating therapeutic applications and functional genomic studies.

Gene editing is the term used to describe the precise modification of nucleic acid sequences. The CRISPR/Cas9 system has transformed this field by facilitating the efficient and programmable modification of genetic and nongenetic diseases. Methods to detect and improve the precision of CRISPR/Cas9 have been developed in response to concerns regarding off-target effects.^135^ The CRISPR/Cas gene-editing system exhibits potential for genome manipulation; however, it encounters obstacles in terms of intracellular delivery efficacy for clinical applications. Liposomes, polymers, and nanoparticles are examples of nanocarriers that can potentially improve therapeutic applications.^136^

#### 6.3.3 RNA Sequencing (RNA-Seq)

Assists in comprehending gene function and regulation by measuring gene expression levels across the entire transcriptome.

The significant advancements in RNA sequencing have given rise to an essential technique for transcriptome profiling. The transition from bulk RNA sequencing to more advanced techniques such as single-molecular, single-cell, and spatial transcriptome methods has revolutionized how we incorporate spatial data. These methods now enable us to focus on individual cells with higher accuracy. Medical research and clinical therapy face significant challenges when it comes to cancer, a widespread and complex disease with devastating consequences.^137^ In recent years, RNA sequencing has stimulated a substantial number of research domains. During the reverse transcription reaction, the majority of protocols depend on the synthesis of a more stable complementary DNA (cDNA) copy of the RNA molecule.^138^

#### 6.3.4 Microarrays

Enable the analysis of genetic data on a large scale by detecting gene expression patterns and genetic variations. Protein microarrays are a valuable tool in cancer research due to their significant potential, capacity to handle large amounts of data, immediate display of results, increased sensitivity, minimal sample requirement, and improved adaptability. One study primarily explores the research advancements made in four different types of protein microarrays: proteome microarray, antibody microarray, lectin microarray, and reversed protein array. The focus is particularly on their use in cancer research.^139^ Antibody microarrays have the potential to be of considerable value in biological research. The distinctive experimental capabilities of this technology could be particularly advantageous for cancer research. The direct labeling method, which involves chemically tagging all proteins in a complex mixture, enables the detection of bound proteins after incubation on an antibody microarray. The sandwich test utilizes a combination of antibody detection to identify proteins that have been captured on an antibody microarray. Every antibody is matched with one of the spotted antibodies.^140^

#### 6.3.5 Single-Cell Genomics

**Figure 5:**
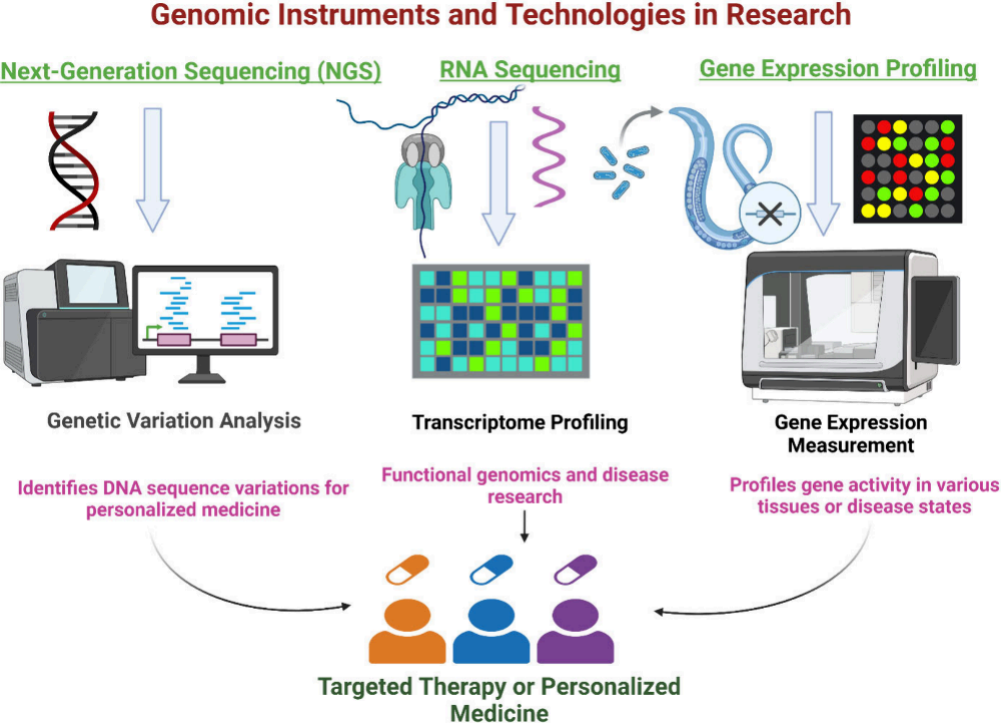
Genomic instruments and technologies in research. The illustration emphasizes essential approaches including next-generation sequencing, RNA sequencing, and gene expression profiling. These tools provide comprehensive study of genetic variations, functional genomics, and targeted therapies, propelling progress in personalized medicine and enhancing our comprehension of the genome in contemporary research.

Investigates cellular heterogeneity and intricate biological processes by analyzing genetic information at the single-cell level. The advancement of single-cell technology has significantly enhanced research on cardiovascular diseases (CVDs). These single-cell technologies have found extensive use in studying various cardiovascular conditions, including atherosclerosis, myocardial infarction, cardiac ischemia-reperfusion injury, arrhythmia, hypertrophic cardiomyopathy, and heart failure. They offer valuable insights into the fundamental processes of cardiovascular disease, exploring its different aspects at the DNA, RNA, protein, post-transcriptional, post-translational, and metabolite levels.^141^ The various genomic instruments and technologies in research has been listed out in Figure 5 and summary of genomic investigation on hepatoprotective compounds in Table 6.

**Table 6:** Summary of Key Genomic Studies on Hepatoprotective Agents

S.N.	Hepatoprotective Agent	Genomic Findings	Implications	References
1	Silymarin	Modulation of gene expression related to oxidative stress and inflammation	Indicates silymarin’s role in enhancing antioxidant defenses and reducing inflammatory responses	Macit, M., Duman, G.^142^
2	Curcumin	Upregulation of Nrf2 and downstream antioxidant genes	Supports curcumin’s potential in protecting liver cells from oxidative damage	Huminiecki, L., Horbańczuk, J., Atanasov, A.^143^
3	Glycyrrhizin	Downregulation of pro-inflammatory cytokine genes	Highlights glycyrrhizin’s anti-inflammatory effects at the genomic level	Salminen, H., Kasapoğlu, K.^56^
4	Andrographolide	Modulation of genes involved in apoptosis and cell survival	Suggests andrographolide’s ability to promote cell survival and prevent apoptosis in hepatocytes	Jain, K., Majee, C.^144^
5	Quercetin	Influence on the expression of genes related to lipid metabolism and oxidative stress	Indicates quercetin’s role in regulating lipid metabolism and protecting against oxidative stress	Costa, A., de Sousa, L.^81^
6	Picroside	Activation of genes involved in liver regeneration and repair	Demonstrates picroside’s potential in promoting liver regeneration and healing	Gaikwad, P., Bhope, S.^58^
7	Phyllanthin	Modulation of detoxification genes	Highlights phyllanthin’s role in enhancing liver detoxification processes	Nasrulloh, R., Rafi, M.^60^
8	Berberine	Regulation of genes involved in lipid metabolism and inflammatory pathways	Supports berberine’s beneficial effects on lipid metabolism and its anti-inflammatory properties	Sardana, S., Gupta, R.^145^
9	Ginsenosides	Influence on genes related to oxidative stress, inflammation, and apoptosis	Indicates ginsenosides’ multifaceted role in liver protection	Ratan, Z., Haidere, M.^146^
10	Oleanolic Acid	Upregulation of antioxidant genes and downregulation of fibrogenic genes	Suggests oleanolic acid’s potential in preventing liver fibrosis and enhancing antioxidant defenses	Castellano, J., Ramos-Romero, S., Perona, J.^147^

### 6.4 Examples of Genomic Studies on Natural Hepatoprotective Agents

#### 6.4.1 Silymarin (Milk Thistle)

The advancements in molecular markers and genomic research in milk thistle are greatly restricted. The collection includes 220 milk thistle resources, with 172 accessions obtained from the domestic market and 48 from 6 accessions distributed by the National Agrobiodiversity Center in Korea. Recordings were taken for six plant attributes: height, seed weight, flower count, seed weight per flower, spine length, and hue at harvest^148^

#### 6.4.2 Curcumin (Turmeric)

Curcumin, a naturally occurring plant substance, has sparked significant interest due to its hepatoprotective properties. Genes that are expressed differently have been associated with the control of the cell cycle, programmed cell death, and cellular communication. Curcumin has been found to have a significant impact on microRNA production, which plays a crucial role in regulating cancer growth and can lead to specific changes in methylation.

#### 6.4.3 Resveratrol (Grapes)

*Alternaria sp*. MG1, a fungus commonly found inside grapes, can naturally produce resveratrol, a chemical compound with great potential for various applications. Nevertheless, there is still much to discover about the metabolic traits and physiological actions of MG1. Furthermore, the strain only produces small amounts of resveratrol. Therefore, comprehending the resveratrol production process hinges on the heightened need for whole-genome sequencing^149^

#### 6.4.4 Glycyrrhizin (Licorice)

Liquorice root contains several enzymes and transporters which play a major role in forming glycyrrhizin. Enzymes in *G. uralensis* are responsible for forming isoflavonoids.^150^

#### 6.4.5 Quercetin (Various Fruits and Vegetables)

Quercetin shows therapeutic benefits in hepatic disorders by regulating the expression of genes associated with inflammation, cell proliferation, and ECM modeling^151^

#### 6.4.6 Berberine (Berberis Species)

Berberine has the potential in the treatment of alcoholic fatty liver. Genomic studies have supported this by elucidating the potential of berberine in regulating the expression of genes involved in lipid metabolism, insulin signaling, and inflammation.

#### 6.4.7 Epigallocatechin-3-gallate (EGCG) (Green Tea)

Studies on EGCG showed its potential in the expression of genes involved in lipid metabolism, inflammation, and antioxidant defense and helps in the understanding of how EGCG protects against alcohol-induced liver damage.^152^

#### 6.4.8 Andrographolide (Andrographis paniculata)

Research data shows that andrographolide has the potential to regulate genes associated with inflammation, oxidative stress, and cell survival and exert hepatoprotective effects.

## 7 Synergistic Effects and Combinatorial Strategies

Synthetic pharmaceuticals, including silymarin, pentoxifylline, and ademetionine, are frequently employed for hepatoprotection. Nonetheless, inadequate absorption and adverse effects frequently constrain their therapeutic efficacy. Incorporating these with herbal extracts, genomics, and nanotechnology can improve efficacy by addressing numerous pathways, such as oxidative stress, inflammation, and apoptosis. Integrating herbal-based extract with these conventional pharmaceuticals, genomics, and nanotechnology can produce synergistic effects by targeting multiple pathways like oxidative stress, inflammation, and apoptosis, thereby providing a comprehensive hepatoprotection.

### 7.1 Combining Natural Products with Synthetic Drugs

Silymarin is a flavonoid and is known for its hepatoprotective activity due to its high antioxidant potential. However, its clinical efficiency remains challenging, as the compound exhibits poor bioavailability. The efficacy of silymarin when administered with natural bioenhancers like piperine, fluvic acid, and lysergol in CCl_4_-induced hepatotoxicity was studied, where the use of bioenhancers improved the bioavailability of silymarin., and showed enhanced hepatoprotectivity.^153^ Silymarin and synthetic derivatives of anthranilic acid, azomethines, and alkyl-2-sterylquinolic acid were administered, improving silymarin’s therapeutic effect.^154^

### 7.2 Synergistic Effects of Multiple Natural Products

Natural substances, like flavonoids and polyphenols, frequently have combinatorial actions that augment hepatoprotection. Combining isoflavones and curcumin has significant antioxidant and anti-inflammatory properties, mitigating liver fibrosis and oxidative stress. Moreover, bioactive chemicals in dietary sources interact intricately, affecting their therapeutic effectiveness.^155^ Considering the significant health advantages they offer, such as reducing the risk of chronic diseases like cancer, maintaining liver health, preventing cardiovascular issues, and managing type 2 diabetes, it is recommended to regularly incorporate complete meals and a diverse selection of nutritious foods into your diet. Research involves analyzing various models of antioxidants and exploring how they interact in both controlled laboratory settings and living organisms. Additionally, it delves into the effects of phytochemicals in food, specifically examining their synergistic and antagonistic impacts. The main focus is on understanding the biological molecular mechanisms behind these phytochemicals.^156^

**Figure 6:**
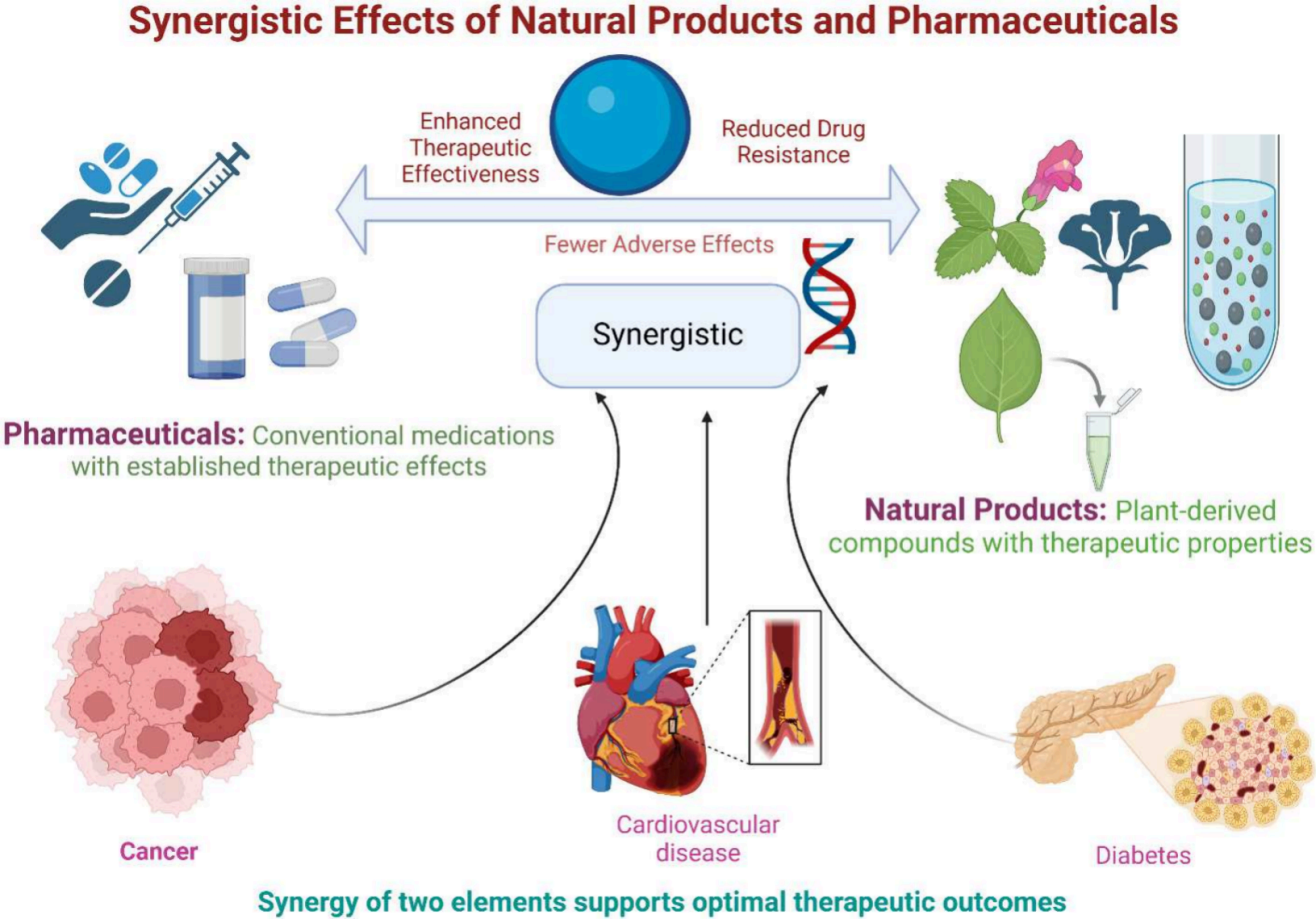
Synergistic effects of natural products and pharmaceuticals. The diagram demonstrates that the amalgamation of natural compounds with traditional pharmaceuticals improves therapeutic effectiveness, diminishes drug resistance, and lessens adverse effects. Case studies demonstrate enhanced results in the management of chronic illnesses, emphasizing the potential of these synergistic interactions to attain optimal therapeutic advantages in treatment protocols.

Various chemicals found in nature can affect multiple targets, resulting in a range of effects that can be additive, synergistic, or antagonistic. To develop medications that are both more effective and safer, it is crucial to assess the potential various effects of natural products thoroughly. To comprehensively assess potential synergistic effects, computational techniques like PASS software can forecast pharmacotherapeutic interactions. PASS evaluates drug-like compounds, examining more than 3,500 effects, such as hepatoprotective capacity, metabolic interactions, and toxicity. This method facilitates the identification of appropriate combinations of natural and synthetic drugs.^157^ The synergistic effects of natural products and pharmaceuticals shown in Figure 6.

### 7.3 Case Studies of Successful Combinatorial Approaches

Herbal remedies have been employed for a variety of purposes. Safety is one of the concerns associated with herbals, given the diverse uses of these materials. It is widely recognized that contemporary medications can induce severe adverse effects. Latrogenic diseases are the fourth most common cause of mortality in the United States and other developed countries. Nootropics, antidiabetics, hepatoprotective, and lipid-lowering agents are promising pharmacological agents. Herbal medications are frequently the sole viable alternative in rural areas. It is less expensive than contemporary medicine, which also necessitates extensive research. Modern medicine is unable to provide alleviation to the average person.^158^ Drug-induced liver injury is a common and serious health concern that can have a negative impact on disease treatments. At present, there are no specific clinical medications accessible for drug-induced liver injury. Traditional natural remedies have been widely used as health products. Certain natural remedies have been found to possess hepatoprotective properties, showing minimal adverse reactions and significant clinical effectiveness. Thus, natural remedies show potential as a viable method for addressing drug-induced liver injury.^159^

Acetaminophen significantly contributes to drug-induced liver impairment, especially when used in conjunction with hepatotoxic agents such as antituberculosis treatments. It is a major contributor to liver damage caused by drugs, especially when used alongside antituberculosis medications. Scientists have conducted thorough investigations on natural products to assess their potential to mitigate liver damage caused by acetaminophen. They help to reduce mitochondrial dysfunction and inflammation, prevent oxidative/nitrative stress, and protect against macromolecular damage, showing significant benefits for liver health. Using natural products as an adjuvant with existing medications or as a standalone treatment is promising due to their bioavailability and dietary nature.^160^ Polyherbal capsules are commonly prescribed to help protect the liver and treat conditions such as alcoholic liver disease and hepatitis. The product contains a potent blend of natural ingredients, carefully selected and scientifically proven for their beneficial properties. The formula includes a concentrated form of Bhumyamalaki, Liquirice, Punarnava, Bhringraj, Tulsi, Daruharidra, and Pippali. On an individual basis, these substances have demonstrated their ability to protect the liver. The study examined the hepatoprotective effects of Polyherbal Capsules compared to other products available. The research focused on ethanol-induced liver damage in rodents^161^ The summary of case studies with strategy, findings and implications listed in Table 7.

**Table 7:** Summary of Case Studies with Successful Combinatorial Strategies

S.N.	Combinatorial Strategy	Findings	Implications	References
1	Silymarin and Curcumin	Enhanced antioxidant and anti-inflammatory effects, reduced liver enzymes, and improved histopathology	Combination therapy offers superior protection against liver damage compared to individual agents	Korany, M., Haggag, R^162^
2	Glycyrrhizin and Andrographolide	Synergistic reduction in inflammation markers and liver fibrosis, improved liver function	Highlights the potential for using combined natural products for managing liver fibrosis	Dash, R., Kala, M.^163^
3	Quercetin and Resveratrol	Improved mitochondrial function, decreased oxidative stress, and enhanced cell survival in hepatic cells	Supports the use of flavonoid combinations for better mitochondrial protection and liver health	Alam, S., Wagner, A.^164^
4	Picroside and Phyllanthin	Enhanced liver regeneration, reduced oxidative damage, and improved liver enzyme levels	Demonstrates the effectiveness of combining hepatoprotective agents for liver repair and recovery	Shanbhag, S., Bachute, M.^165^
5	Berberine and Ginsenosides	Significant reduction in lipid accumulation, improved insulin sensitivity, and decreased liver inflammation	Effective for managing nonalcoholic fatty liver disease and associated metabolic issues	Xu, L., Zhao, W.^166^
6	Curcumin and Piperine	Increased bioavailability of curcumin, enhanced anti-inflammatory and antioxidant effects	Piperine enhances the efficacy of curcumin by increasing its bioavailability	Baspinar, Y., Üstündas, M.^167^

## 8 Clinical Studies and Human Trials

Hepatoprotective activity is the primary focus of clinical studies and human trials, which assess the effectiveness of natural compounds in safeguarding the liver. These studies evaluate biomarkers, liver function tests, and histopathological alterations, thereby substantiating the therapeutic potential of these compounds in liver diseases.

### 8.1 Overview of Clinical Trials on Natural Hepatoprotective Agents

To comprehend the hepatoprotective benefits demonstrated in clinical studies, it is essential to investigate the bioactive molecules accountable for these therapeutic outcomes. The primary bioactive molecules contributing to the hepatoprotective effect are secondary metabolites, including alkaloids, flavonoids, phenolics, tannins, lignins, and resin-based compounds. The study centered on the evidence and mechanism of hepatoprotection shown by impure extracts of medicinal herbs. Many plant extracts have a significant impact on the body by neutralizing harmful free radicals that are produced during various diseases. Numerous scientific studies on living organisms have revealed the significant impact of phenolic and flavonoid components found in plant extracts. These components have been found to increase blood glutathione levels, stimulate protein secretion, reduce lipid peroxidation, and enhance the ability to eliminate free radicals. The efficacy of these natural compounds from plants has been scientifically proven to treat obesity, diabetes, renal disease, and cardiovascular disease. Caffeine and catechins in green tea have been found to reduce body mass index and waist circumference. The cocoa’s catechins, anthocyanins, and proanthocyanidins reduce blood pressure and blood glucose levels. Serval herbal compounds like curcumin, resveratrol, ginkgo biloba, and silymarin offer hepatoprotective activity.^168^ This is due to their ability to reduce factors like cytokines and a wide range of enzymes.^169^ Curcumin is a naturally occurring phenolic compound found in several plants, with turmeric being the major source. It was found that this compound processes anti-inflammatory, anticancer, antioxidant, and neuroprotective activities in both lab animals and human participants. Further, it was also reported to have antidiabetic, antirheumatoid, and anticoagulant activity. Flavonoids are dietary bioactive compounds that are naturally derived from plants and have a profound influence on human health. Morin hydrate is derived from the fruits, stems, and foliage of plants in the Moraceae family. The extensive evidence available strongly supports the positive impact of Morin hydrate on a wide range of chronic and life-threatening degenerative diseases.^170^

**Figure 7:**
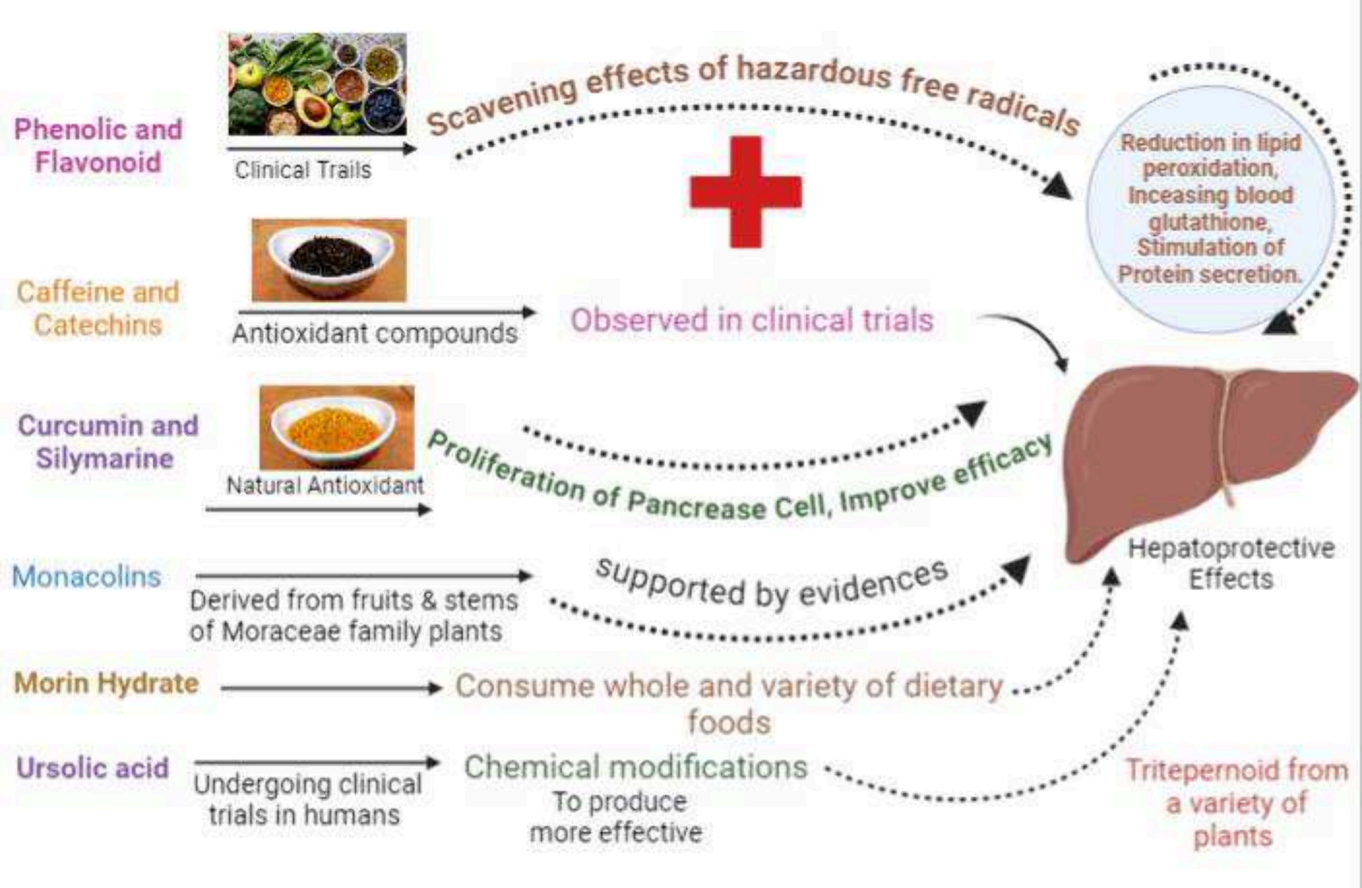
Clinical trial procedure for hepatoprotective pharmaceuticals. The diagram delineates essential phytoconstituents preclinical research, safety evaluation, efficacy examination, and large-scale assessment. It encompasses participant recruiting, dosage optimization, and hepatic function assessment. This procedure guarantees a thorough evaluation of novel hepatoprotective treatments to verify their safety and efficacy for clinical application.

Human clinical trials are currently underway to test the efficacy of ursolic acid in treating tumors, cancer, and skin wrinkles. Ursolic acid has been utilized in clinical settings to address a range of ailments, including liver protection and combating cancer. Furthermore, it delves into the isolation and purification of this triterpenoid from different plant sources to enhance our understanding of the practical application of analytical techniques for ursolic acid analysis. Furthermore, the technique of altering the chemical composition of ursolic acid to create derivatives with enhanced efficacy and water solubility, along with the existing understanding of its partially synthetic and natural counterparts^171^ The clinical trial procedure for hepatoprotection active pharmaceuticals shown in Figure 7.

### 8.2 Challenges in Translating Preclinical Findings to Clinical Settings

Often necessitating extensive and costly clinical trials for validation, the translation of preclinical findings to clinical settings is impeded by various factors, including differences in species biology, variability in drug metabolism, inadequate administration strategies, and the assurance of human safety and efficacy. Acetaminophen hepatotoxicity is exacerbated by endoplasmic reticulum stress. Guanabenz, an antihypertensive drug, reduces the formation of toxic metabolites, enhances the production of nontoxic metabolites, and mitigates ER and oxidative stress. It exhibits potential as a hepatoprotective treatment and maintains analgesic efficacy when combined with acetaminophen. These results offer preclinical evidence that GA is a promising antidote for the treatment of APAP-induced liver toxicity and suggest that it may be combined with APAP in clinical settings.^172^ Although synthetic chemicals such as Guanabenz exhibit potential, the sustainability of natural hepatoprotective substances remains a vital factor in therapeutic development. Rapid reduction in the natural resources due to excessive wastage can also pose a challenge for translation; *Abelmoschus manihot*, a flowering perennial plant, is amino acids, flavonoids, organic acids, polysaccharides, and volatile compounds, is already in rapid reduction stages.

The precise bioactive compounds and the underlying mechanisms of action of the blooms have yet to be fully comprehended or elucidated at present. A thorough examination of the practical uses, chemical makeup, effects on the body, and various aspects related to the development and use of *A. manihot* provides a solid foundation for future scientific progress.^173^

The herbal mixture that is employed to promote liver health and contains methanol extracts of *Ruta graveolens* and *Angelica sinensis*. Evaluations indicated that these possesses robust antioxidant properties, which are indicative of its ability to mitigate oxidative stress and neutralize hazardous free radicals. Additional research in the clinical setting and other fields of study may provide a more comprehensive comprehension of its applications in promoting liver health and overall well-being.^174^ Dandelion is a widely used hepatoprotective plant in a variety of medicinal systems. The lipogenesis effects of dandelion are linked to the reduction of inflammation in the body and liver, as well as the enhancement of insulin resistance and antioxidant status. The hepatoprotective effects of dandelion have been confirmed by various documents. In order to further evaluate the hepatoprotective effects of dandelion, it is necessary to prepare standard extracts of dandelion with high concentrations of effective compounds and to devise large clinical studies using these extracts.^175^

The protective effects of natural substances on the liver are commonly linked to their ability to act as antioxidants and enhance the body’s own antioxidant defense system. Despite the significant hepatoprotective benefits observed in animal and cell culture models, the lack of clinical research has hindered the formal acceptance of these substances by medical professionals and clinicians. Thus, it is essential to carry out controlled clinical trials to validate the therapeutic efficacy of substances that may have hepatoprotective properties.

Understanding the principles behind the hepatoprotective effect of phytochemicals can play a crucial role in preventing clinical trial failures and informing the development of future medications. Fatigue and abdominal discomfort are frequently observed as symptoms of liver dysfunction, which poses a challenge to the pharmaceutical industry and healthcare. Elevated liver enzymes were observed in 28% of obese patients (BMI > 25). Hepatoprotective effects were demonstrated by Jawarish bisbasa, which was tested on 23 patients, resulting in a significant reduction in liver enzymes.^176^

### 8.3 Success Stories and Ongoing Trials

Cinnamic acid and its derivatives are a group of unsaturated compounds that contain a carboxyl group. Current marketed preparations contain cinnamic acid as the primary moiety and ongoing clinical trials of cinnamic acid derivatives.^177^ Saponins are a well-known group of surfactants. When considering the formulation of these substances as an active ingredient or their use in combination with other medications, it is crucial to take into account the impact of their surface activity on their efficacy and safety. Dai and his associates explored the hepatoprotective effects of *Diammonium glycyrrhizinate* and baicalin. *Diammonium glycyrrhizinate* is known for its hepatoprotective properties and also its biosurfactant effects. Baicalin is also known for its hepatoprotective effects. Experimental results showed that the combinatorial effect of these substances showed improved hepatoprotective effects in CCl4-induced hepatotoxicity toxicity models of rats. when compared to individual substances.^178^ A study of the effect of whole desiccated *Phyllanthus amarus* was conducted on 107 patients with liver disease. At the end of the study, the patients showed a substantial decrease in SGPT and Bilirubin and an increase in hemoglobin levels.^179^ Boldine showed improved hepatoprotective properties in various experimental models, The hepatoprotective effects of Boldine can be attributed to its antioxidant and mitochondrial protective properties.^180^ Existing clinical evidence also shows the use of hepatoprotective agents by pregnant women with impaired cholestasis. These hepatoprotective agents are commonly used in the treatment of drug-induced liver impairment, nonalcoholic fatty liver disease, and hepatitis. These agents act by detoxification, anti-inflammation, antioxidation, and hepatocyte membrane protection., and are effective and safe.^181^ Medicinal mushrooms have shown various pharmacological effects like anti-inflammatory, antimicrobial, antiviral, antidiabetic, antihyperlipidemic, digestive, and hepatoprotective effects. These properties provide significant health benefits. One study presents the effects and mechanisms of bioactive compounds in clinical investigations carried out in laboratory settings (in vitro) and in living organisms (in vivo) for a specific group of medicinal mushrooms.^182^ Liv.52 is a polyherbal composition utilized in India and various other countries for over five decades. Research findings from both preclinical and clinical studies provide strong support for using symptomatic improvement and supportive treatment in addressing a range of liver conditions. These conditions encompass hepatitis (including Hepatitis B), alcoholic liver disease, nonalcoholic fatty liver disease, nonalcoholic steatohepatitis, and hepatotoxicity caused by drugs used in tuberculosis treatment. Liv.52 is an example of a scientific agent^183^ The list of key clinical trials involving natural hepatoprotective agents shown in Table 8.

**Table 8:** Summary of Key Clinical Trials Involving Natural Hepatoprotective Agents

S.N.	Natural Hepatoprotective Agent	Study Design	Key Findings	References
1	Silymarin	Randomized, double-blind, placebo-controlled	Significant reduction in liver enzymes (ALT, AST) and improved liver function in chronic hepatitis C patients	Javed, S., Ahsan, W., Kohli, K.^153^
2	Curcumin	Randomized, controlled trial	Reduced liver enzyme levels and improved liver function tests in patients with nonalcoholic fatty liver disease	Naksuriya, O., Okonogi, S.^118^
3	Glycyrrhizin	Double-blind, placebo-controlled study	Decreased liver fibrosis and improved liver function in patients with chronic hepatitis B	Salminen, H., Kasapoğlu, K.^56^
4	Quercetin	Pilot study, open-label	Decreased oxidative stress and liver enzymes, improved liver histology in patients with alcoholic liver disease	Costa, A., de Sousa, L.^81^
5	Phyllanthin	Double-blind, placebo-controlled study	Reduced liver enzyme levels and improved liver function in patients with viral hepatitis	Shanbhag, S., Bachute, M.^165^
6	Berberine	Randomized, controlled trial	Improved insulin sensitivity, reduced liver fat, and decreased liver enzymes in patients with NAFLD	Sardana, S., Gupta, R.^145^
7	Ginsenosides	Double-blind, placebo-controlled	Significant reduction in liver inflammation and improved liver function in patients with liver cirrhosis	Ratan, Z., Haidere, M.^146^

## 9 Future Perspectives and Challenges

The future of hepatoprotective research includes the development of targeted therapies, the integration of nanotechnology, and the comprehension of genetic variations in response to treatments. The challenges involve translating preclinical findings into clinical practice, the assurance of safety and efficacy, and the surmounting of regulatory and economic obstacles to approving novel therapeutics.

### 9.1 Emerging Trends in Hepatoprotection Research

The current trends in drug delivery are in nano forms. The concept of conventional dosage forms has been changed to enhance the drug or active pharmaceutical ingredient’s solubility, stability, permeability, and bioavailability. The liver cells are damaged in various ways by different factors. This required the effective delivery of active moiety to heal the hepatocytes on different sides.

One notable nanoformulation technique is the newly created propolis micelle formulation, which has shown substantial antioxidant protection. Propolis formulation has demonstrated significant potential in offering antioxidant protection against oxidative stress in laboratory and animal toxicity tests. The formulation contains poplar propolis enclosed in micelles formed by a triblock copolymer known as poly(ethylene oxide)-β-poly(propylene oxide)-β-poly(ethylene oxide). The formulation exhibits a compact size (Dh = 20 nm), exceptional colloidal stability, and enhanced solubility in water.

*Propolis micelles* (20 to 100 μg/mL) improved the survival of HepG2 cells *in vitro* when exposed to H_2_O_2_.^184^*Artemisia absinthium* extract-based nanosuspension prepared by antisolvent precipitation technology improved hepatoprotective efficacy and increased bioavailability over coarse extract.^185^ Silymarin nanoparticles were formulated to evaluate their ability to reduce acetaminophen-induced hepatotoxicity in animal models. The formulated silymarin nanoparticles were observed to have a spherical shape, particle size range of 138.9 to 155 nm, with a zeta-potential of −0.0340, average loading efficiency of 32 ± 0.5%, and improved therapeutic efficiency. A remarkable gift to humanity is the ayurvedic plant *Emblica officinalis (E. officinalis*), which contributes to improving people’s well-being. The medicinal and nutritional benefits of this are significant. Using nanoformulation in *E. officinalis* enhances the release of active components and food ingredients, leading to improved bioaccessibility, enhanced therapeutic effects, and easier digestion within the human body.^186^ Using a specifically created liposomal nanoformulation containing perillaldehyde increased the drug’s effect on elevated cholesterol brought on by poloxamer 407.^187^

**Figure 8:**
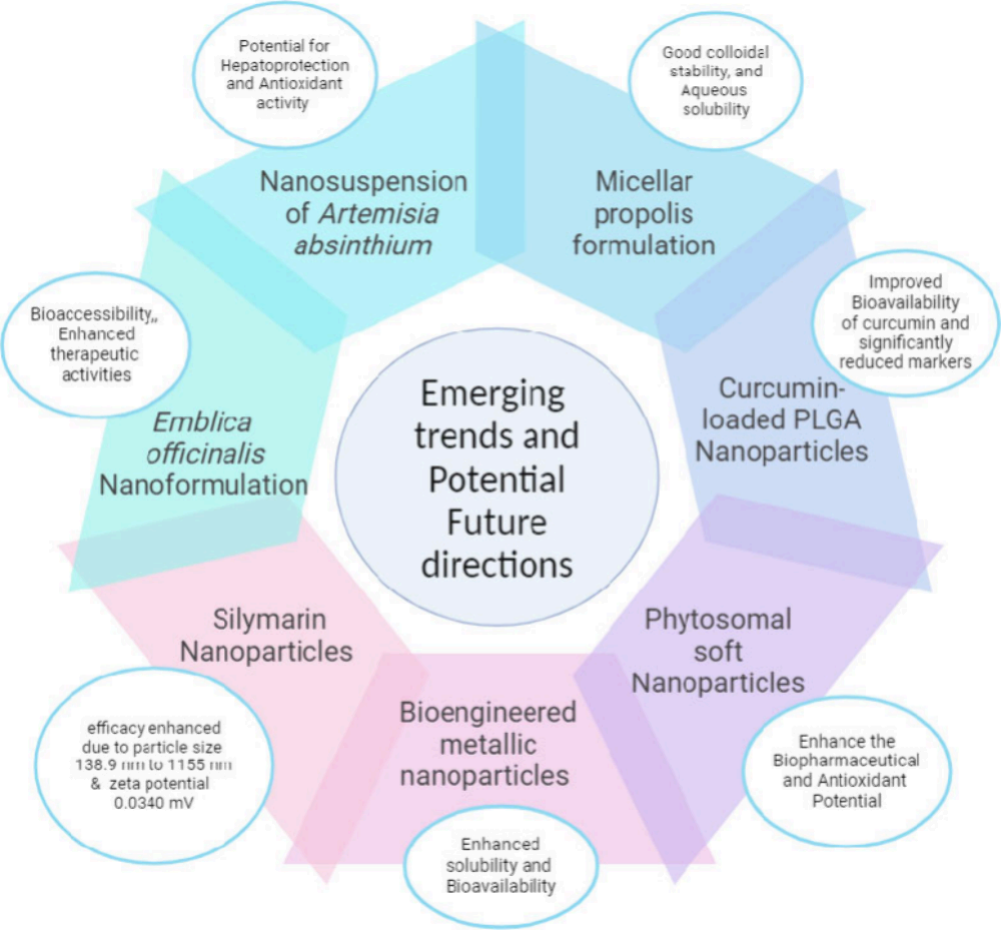
Emerging trends in hepatoprotective research. The figure emphasizes progress in precision medicine, omics technologies, and novel delivery strategies. It investigates molecular pathway elucidations, targeted pharmacological development, and the prospects of innovative natural substances. Future research focuses on tailored treatments, improvements in nanotechnology, and the identification of novel therapeutic molecules, thereby influencing the future of hepatoprotection.

Besides their antibacterial qualities, bioengineered metallic nanoparticles are being investigated for their hepatoprotective potential owing to their antioxidant and anti-inflammatory characteristics. The current state and potential of naturally occurring bioengineered metallic nanoparticles as potent antibacterial agents in the future. The increasing fascination with treating microbial diseases stems from the remarkable characteristics of metallic nanoparticles and the plentiful availability of natural resources.^188^ Solvent evaporation and nanoprecipitation procedures improved the biopharmaceutical and antioxidant characteristics of self-assembled phytosome soft nanoparticles encapsulated with a phospholipid complex (MPLC SNPs). Several methods, such as DSC, TGA, FT-IR, PXRD, 1H-NMR, solubility, in vitro dissolution, oral bioavailability, and in vivo antioxidant tests, were used to characterize MPLC and MPLC SNPs. MPLC SNPs had an almost 10-fold increase in oral bioavailability, which increased Cmax, Tmax, and AUC. Consequently, MPLC SNPs can function as a nanovesicle delivery method, enhancing MGN’s biopharmaceutical and antioxidant qualities.^189^ Few studies reported to provide better therapeutics in hepatoprotective action depicted in Figure 8.

### 9.2 Potential Challenges and Limitations

Notwithstanding the progress in nanoformulations for hepatoprotection, other obstacles persist, such as medication solubility, stability, and targeted delivery. Various drugs are very important for managing various diseases and disorders. Their challenges and limitations include aqueous solubility, intestinal permeability, and stability. To overcome these hurdles, researchers should consider designing the dosage form to ensure its effectiveness, bioavailability, and stability profile.

Isoniazid (INH) is a first-line chemotherapeutic substance that is used to treat tuberculosis. Nevertheless, its medical application was restricted by its limited oral bioavailability, short life, and extensive first-pass metabolism. The objective of the calcium ion-alginate-piperine microspheres (INH-CaSP Ms) is to enhance the effectiveness of INH encapsulation, regulate its release, and enhance its oral bioavailability.^190^ To enhance the oral bioavailability and antioxidant potential of firulic acid, a novel nanocarrier system of chitosan nanoparticles laden with phospholipids complex was created. The aqueous solubility, dissolution, and permeation rate were significantly enhanced due to functional characterization investigations.^191^

Hydrochlorothiazide is a first-line drug that is employed to treat hypertension with limited oral bioavailability as a result of poor aqueous solubility and permeability. Therefore, a solid dispersion based on lyophilized egg white protein was created to investigate its potential as a solid dispersion carrier for improved aqueous solubility and permeability.^192^ Nanotechnology techniques offer a solution to low water solubility in medications classified as Class II and IV in the Biopharmaceutical Classification System. Decreasing the size of medicine particles to the nanoscale is crucial in enhancing a medication’s bioavailability or its dissolution rate.^193^

Nucleic acid therapeutics are costly and have presented substantial stability and delivery challenges. A valuable resource for formulation specialists familiar with the stability profile, regulatory acceptance, and delivery challenges of nucleic acid therapeutics is generated by reviewing and collating the relevant facts and figures because of the limited information available.^194^ Nanotechnology is progressing toward future objectives in medicine, cosmetics, and hospitality due to reducing material size within the 1–100 nm range, improving the material’s bioavailability and stability.^195^

### 9.3 Future Directions for Integrating Nanotechnology and Genomics

The amalgamation of nanotechnology and genomics is set to transform hepatoprotection through the facilitation of individualized medical strategies customized to distinct genetic profiles and metabolic reactions. Personalized medicine is the cornerstone of precision medicine, an innovative medical approach that integrates extensive data analysis, bioinformatics engineering, and genetics. It has undergone significant growth and garnered more interest. Advanced medical technologies are utilized in precision medicine to provide tailored care for specific patients and conditions.^196^ Effective tools, methods, and methodologies that can be applied in a clinical setting are crucial for adapting to the evolving characteristics of organisms.

Incorporation of multidisciplinary infrastructures can aid in the integration of routine clinical practice and precision medicine and can contribute to advanced medical treatments.^197^ The increase in advancements in biotechnology, genetics, and nanotechnology, as well as growing trends in interdisciplinary collaborations, provide compelling reasons for legislative changes in integrating the realms of science, society, and research.^198^ The recent integration of salivary diagnostic technology and salivary proteosome examination can potentially investigate oral and systemic disorders.^199^ Microchip electrophoresis, based on microfluidization technology, is used in the field of genomics to analyze DNA samples. This offers potential benefits over traditional methods in smaller sample sizes and dimensions.^200^ Advanced biotechnology methods, proteomics, and metabolomics have shown groundbreaking self-quantification and personal genomics applications. These advancements further lead to newer postgenomic innovations converging with nanotechnology.^201^ The huddle possibilities and their solutions have been listed in Table 9.

**Table 9:** Summary of Challenges and Proposed Solutions in the Field

S.N.	Challenge	Description	Proposed Solutions	References
1	Limited Bioavailability	Many natural hepatoprotective agents have poor absorption and bioavailability.	Develop advanced delivery systems (e.g., nanoparticles, liposomes) to enhance bioavailability.	Manach, C., Scalbert, A.^111^
2	Lack of Standardization	Variability in the quality and concentration of active compounds in natural products.	Implement standardized extraction and formulation processes, and establish quality control protocols.	Ulusoy, A., Ela, M.^202^
3	Safety and Toxicity Concerns	Potential for adverse effects or interactions with other medications.	Conduct thorough safety and toxicity studies, and monitor for drug interactions	Soleimani, V., Delghandi, P.^55^
4	Inconsistent Clinical Evidence	Limited and sometimes conflicting evidence from clinical trials.	Design well-structured, large-scale, and long-term clinical trials to generate robust data.	Kantharia, C., Kumar, M.^183^
5	Regulatory Hurdles	Challenges in regulatory approval and market access for natural products.	Work closely with regulatory agencies to meet standards and expedite approvals.	Furfaro, L., Payne, M., Chang, B.^203^
6	High Cost of Development	Expensive process for developing and commercializing new hepatoprotective agents.	Explore cost-effective research methodologies and collaborative partnerships to share development costs.	Mattingly, T., Weathers, S.^116^
7	Intellectual Property Issues	Challenges related to patenting and protecting innovations in natural products.	Seek patents on novel formulations and delivery methods, and explore licensing opportunities.	Hammond, H., Cohen, J.^204^

Nanoformulation with curcumin, such as lipid-based carriers and polymeric nanoparticles, has demonstrated enhanced stability, solubility, and targeting for liver protection. The genomic approach in key polymorphism in genes interfering in liver diseases enhances the development of personalized liver protection strategies. Incorporation of these tools with herbal products explores promising support for the assessment of treatment outcomes and addressing challenges associated with hepatoprotective activity.

## 10 Conclusion

Maladies of the liver are a global concern, and natural products such as curcumin and silymarin exhibit a promising hepatoprotective effect by exerting antioxidant, anti-inflammatory, and detoxifying effects. Several studies reported the hepatoprotective effects of natural products and were validated by clinical trials. However, challenges associated with bioavailability and toxicity have to be addressed. It is essential to understand the cellular and molecular mechanisms underlying hepatotoxicity to develop effective therapeutics. Influence of genetic factors on hepatoprotection of the natural products can be assessed by genomic approach, using advanced technologies such as CRISPR and RNA sequencing. The current review gives an overview of integration genomics, nanotechnology, and mechanistic insights, emphasizing the sophisticated strategies for improving the hepatoprotective effect of natural products. A synergistic effect can also be observed in a combinatorial therapy using natural products with synthetic molecules. To advance further, integration of AI and machine learning, multitargeted approaches, and personalized medicine should be the focus of future research for better therapeutic outcomes.
